# Supplementary material for: Discovery of Indole–Thiourea Derivatives as Tyrosinase Inhibitors: Synthesis, Biological Evaluation, Kinetic Studies, and In Silico Analysis
Source: Int J Mol Sci. 2024 Sep 5;25(17):9636. doi: 10.3390/ijms25179636 (PMC11394742; doi:10.3390/ijms25179636)

Discovery of Indole–Thiourea Derivatives as Tyrosinase Inhibitors: Synthesis, Biological Evaluation, Kinetic Studies, and *In Silico* Analysis

Yang Xu [1], Xuhui Liang[1] and Chang-Gu Hyun [1,2*]

[1] Jeju Inside Agency and Cosmetic Science Center, Jeju National University, Jeju 63243, Korea

[2] Department of Beauty and Cosmetology, Jeju National University, Jeju 63243, Korea

* Correspondence: cghyun@jejunu.ac.kr; Tel.: +82-64-754-1803

Supplementary materials

Table S1. Gradient elution of the mobile phase for the synthetic compounds using HPLC. 4

Table S2. The stability of the synthesized compounds in aqueous solution. 4

Table S3. ADMET properties of the compounds. 9

Table S4. Drug-likeness properties of the compounds. 10

Table S5. Two-dimensional molecular docking plots of the synthesized compounds with mTYR and TYRP1. 10

Table S6. The docking energy of ten repeated molecular dockings of compound **4b** with tyrosinase. 14

Figure S1. The inhibition percentage of DPPH by the synthesized compound. 15

Figure S2. ^{1}H NMR of the 1-ethyl-1H-indole-3-carbaldehyde compound (**2f**). 16

Figure S3. ^{1}H NMR of the 1-methyl-1H-indole-3-carbaldehyde compound (**2g**). 17

Figure S4. ^{1}H NMR of the (E)-2-((1H-indol-3-yl)methylene)hydrazine-1-carbothioamide compound (**4a**). 18

Figure S5. HRMS of the (E)-2-((1H-indol-3-yl)methylene)hydrazine-1-carbothioamide compound (**4a**). 19

Figure S6. ^{1}H NMR of the (E)-2-((5-fluoro-1H-indol-3-yl)methylene)hydrazine-1-carbothioamide compound (**4b**). 20

Figure S7. ^{13}C NMR of the (E)-2-((5-fluoro-1H-indol-3-yl)methylene)hydrazine-1-carbothioamide compound (**4b**). 21

Figure S8. HRMS of the (E)-2-((5-fluoro-1H-indol-3-yl)methylene)hydrazine-1-carbothioamide compound (**4b**). 22

Figure S9. ^{1}H NMR of the (E)-2-((5-chloro-1H-indol-3-yl)methylene)hydrazine-1-carbothioamide compound (**4c**). 23

Figure S10. ^{13}C NMR of the (E)-2-((5-chloro-1H-indol-3-yl)methylene)hydrazine-1-carbothioamide compound (**4c**). 24

Figure S11. HRMS of the (E)-2-((5-chloro-1H-indol-3-yl)methylene)hydrazine-1-carbothioamide compound (**4c**). 25

Figure S12. ^{1}H NMR of the (E)-2-((5-bromo-1H-indol-3-yl)methylene)hydrazine-1-carbothioamide compound (**4d**)........ 26

Figure S13. HRMS of the (E)-2-((5-bromo-1H-indol-3-yl)methylene)hydrazine-1-carbothioamide compound (**4d**)........ 27

Figure S14. ^{1}H NMR of the (E)-2-((5-methoxy-1H-indol-3-yl)methylene)hydrazine-1-carbothioamide compound (**4e**). 28

Figure S15. ^{13}C NMR of the (E)-2-((5-methoxy-1H-indol-3-yl)methylene)hydrazine-1-carbothioamide compound **(4e)**. 29

Figure S16. HRMS of the (E)-2-((5-methoxy-1H-indol-3-yl)methylene)hydrazine-1-carbothioamide compound (**4e**). 30

Figure S17. ^{1}H NMR of the (E)-2-((1-ethyl-1H-indol-3-yl)methylene)hydrazine-1-carbothioamide compound (**4f**)........ 31

Figure S18. ^{13}C NMR of the (E)-2-((1-ethyl-1H-indol-3-yl)methylene)hydrazine-1-carbothioamide compound (**4f**)........ 32

Figure S19. HRMS of the (E)-2-((1-ethyl-1H-indol-3-yl)methylene)hydrazine-1-carbothioamide compound (**4f**)........ 33

Figure S20. ^{1}H NMR of the (E)-2-((1-methyl-1H-indol-3-yl)methylene)hydrazine-1-carbothioamide compound (**4g**)........ 34

Figure S21. HRMS of the (E)-2-((1-methyl-1H-indol-3-yl)methylene)hydrazine-1-carbothioamide compound (**4g**)........ 35

Figure S22. ^{1}H NMR of the (E)-2-((5-methoxy-1-methyl-1H-indol-3-yl)methylene)hydrazine-1-carbothioamide compound (**4h**). 36

Figure S23. ^{13}C NMR of the (E)-2-((5-methoxy-1-methyl-1H-indol-3-yl)methylene)hydrazine-1-carbothioamide compound (**4h**). 37

Figure S24. HRMS of the (E)-2-((5-methoxy-1-methyl-1H-indol-3-yl)methylene)hydrazine-1-carbothioamide compound (**4h**). 38

Figure S25. ^{1}H NMR of the (E)-2-((5-bromo-1-methyl-1H-indol-3-yl)methylene)hydrazine-1-carbothioamide compound (**4i**). 39

Figure S26. ^{13}C NMR of the (E)-2-((5-bromo-1-methyl-1H-indol-3-yl)methylene)hydrazine-1-carbothioamide compound (**4i**). 40

Figure S27. HRMS of the (E)-2-((5-bromo-1-methyl-1H-indol-3-yl)methylene)hydrazine-1-carbothioamide compound (**4i**). 41

Figure S28. ^{1}H NMR of the methyl(E)-3-((2-carbamothioylhydrazineylidene)methyl)-1H-indole-5-carboxylate compound (**4j**). 42

Figure S29. ^{13}C NMR of the methyl(E)-3-((2-carbamothioylhydrazineylidene)methyl)-1H-indole-5-carboxylate compound (**4j**). 43

Figure S30. HRMS of the methyl(E)-3-((2-carbamothioylhydrazineylidene)methyl)-1H-indole-5-carboxylate compound (**4j**). 44

Figure S31. ^{1}H NMR of the (E)-2-((5-hydroxy-1H-indol-3-yl)methylene)hydrazine-1-carbothioamide compound (**4k**). 45

Figure S32. ^{13}C NMR of the (E)-2-((5-hydroxy-1H-indol-3-yl)methylene)hydrazine-1-carbothioamide compound (**4k**). 46

Figure S33. HRMS of the (E)-2-((5-hydroxy-1H-indol-3-yl)methylene)hydrazine-1-carbothioamide compound (**4k**). 47

Table S1. Gradient elution of the mobile phase for the synthetic compounds using HPLC.

The mobile phase consisted of Solution A (0.1% acetic acid in water) and Solvent B (acetonitrile), with gradient elution employed throughout the analysis. The flow rate was set at 2.0 mL/min.

Time	Solution (A) %	Solution (B) %
0 to 10 min	90	10
10 to 20 min	60	40
20 to 28 min	30	70
28 to 30 min	5	95
30 to 40 min	90	10

Table S2. The stability of the synthesized compounds in aqueous solution.

Each compound (2.0 mg) was dissolved in an appropriate volume of water to achieve an approximate concentration of 1-2 mg/mL. The solutions were then subjected to ultrasonic treatment, filtered, and analyzed. During stability testing, varying degrees of crystal precipitation were observed in all samples over time. To facilitate analysis, these samples were reconstituted with additional water or treated ultrasonically, filtered, and then HPLC analysis.

The UV spectra of all synthetic compounds in the mobile phase showed maximum absorption at approximately 275 nm, which was selected for detection. The integration method employed was based on the minimum area approach to accurately determine the retention time (min) and main peak area (%) of all synthesized compounds. The prepared samples were stored at room temperature, and their stability in aqueous solution was evaluated at 0, 2, 4 days, and 2 months using the aforementioned analytical method.

Compounds	0 day		2 day		4 days		2 months	
	RT	Area	RT	Area	RT	Area	RT	Area
4a	21.543	97.61	21.560	95.84	21.618	95.76	21.511	91.27
4b	22.529	97.21	22.518	97.10	22.544	97.07	22.475	91.69
4c	24.104	96.24	24.075	96.04	24.097	95.74	24.057	91.89
4d	24.547	97.79	24.509	97.41	24.518	96.80	24.501	91.28
4e	21.666	98.00	21.684	97.87	21.663	96.87	21.659	94.97
4f	25.863	97.80	25.816	96.99	25.823	96.45	25.795	92.18
4g	24.215	97.47	24.188	94.12	24.194	94.58	24.187	95.20
4h	24.226	96.18	24.178	95.87	24.208	95.83	24.185	90.15
4i	26.507	90.17	26.449	88.52	26.459	88.22	26.453	65.06
4j	21.939	97.37	21.962	97.13	21.973	96.75	21.953	90.86
4k	15.903	96.00	16.117	95.85	16.232	95.32	16.099	94.13

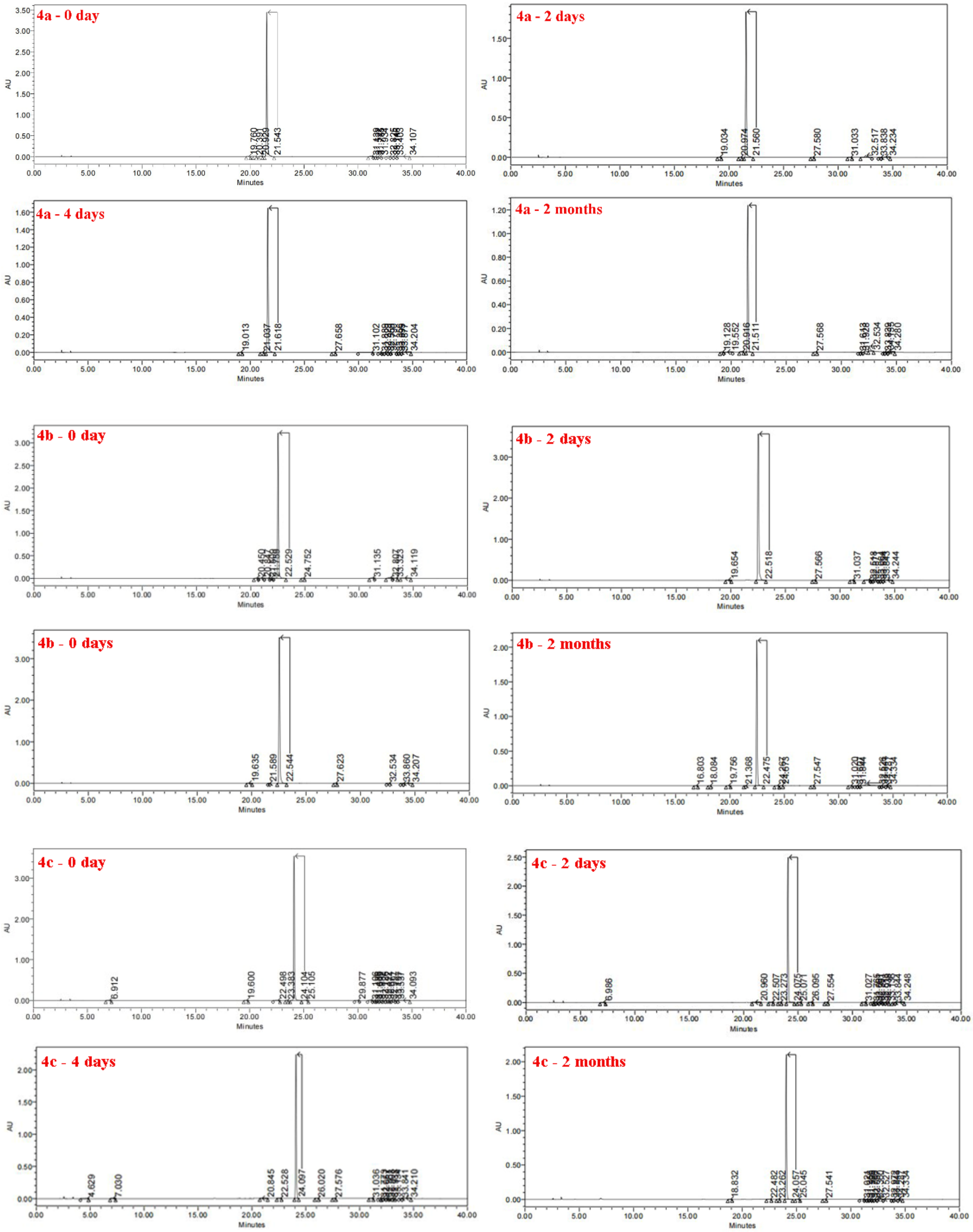
4a - 0 day
4a - 2 days
4a - 4 days
4a - 2 months
4b - 0 day
4b - 2 days
4b - 0 days
4b - 2 months
4c - 0 day
4c - 2 days
4c - 4 days
4c - 2 months
AU
Minutes

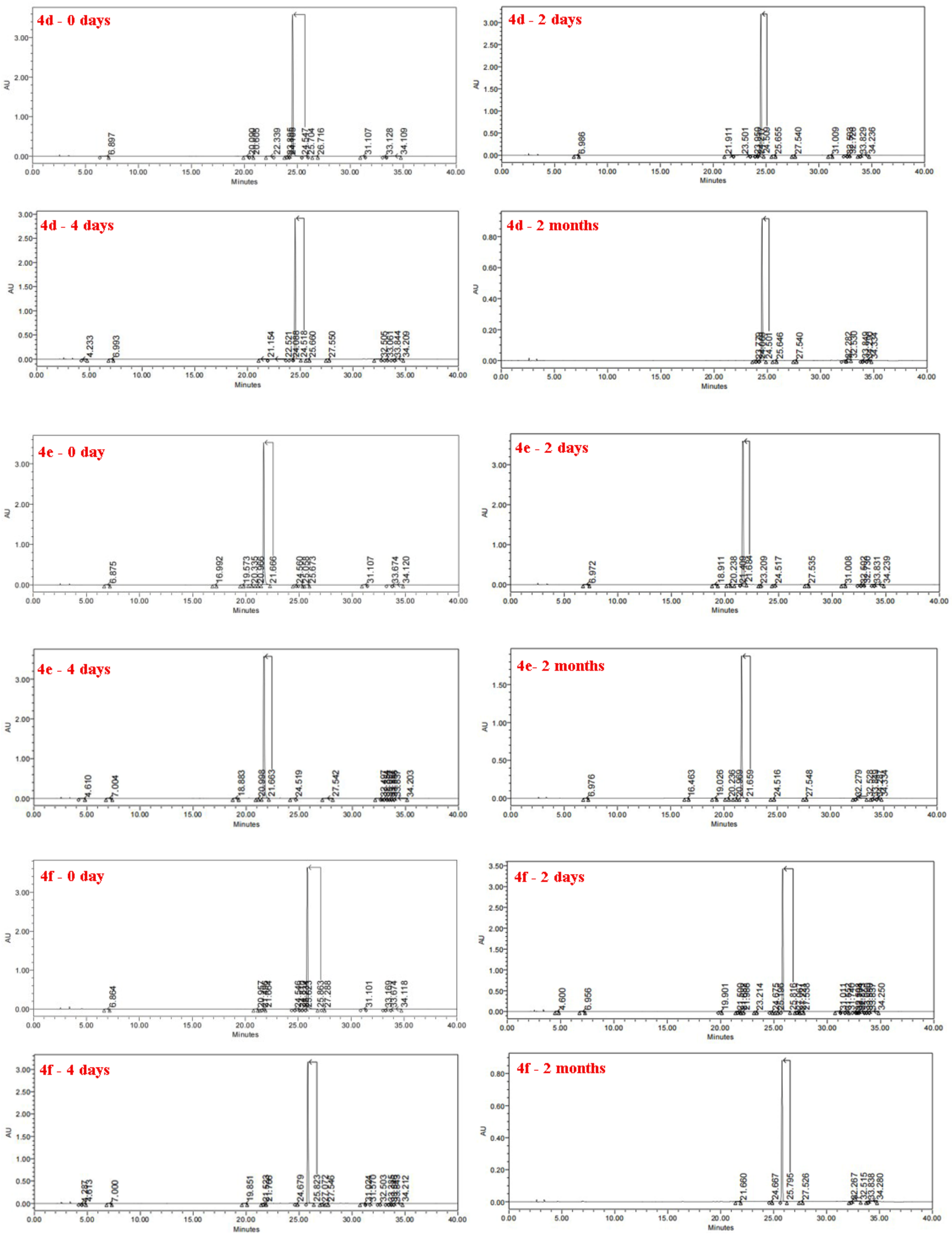
4d - 0 days
4d - 2 days
4d - 4 days
4d - 2 months
4e - 0 day
4e - 2 days
4e - 4 days
4e- 2 months
4f - 0 day
4f - 2 days
4f - 4 days
4f - 2 months
Minutes
AU

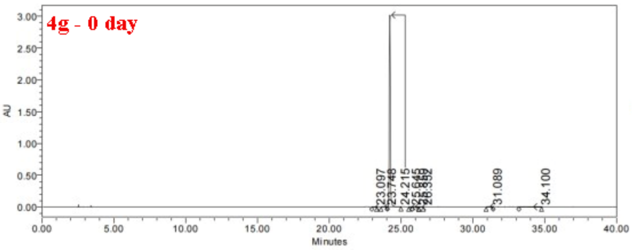
4g - 0 day
AU
Minutes

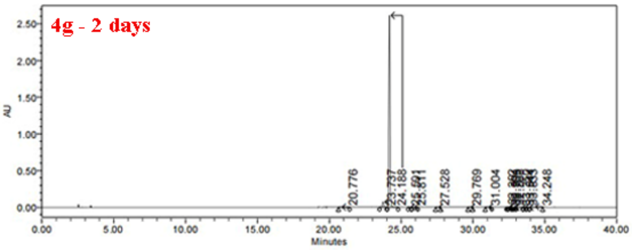
4g - 2 days
AU
Minutes

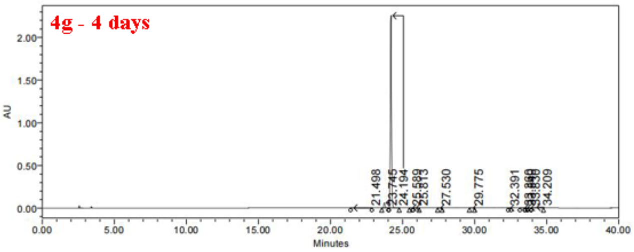
4g - 4 days
AU
Minutes

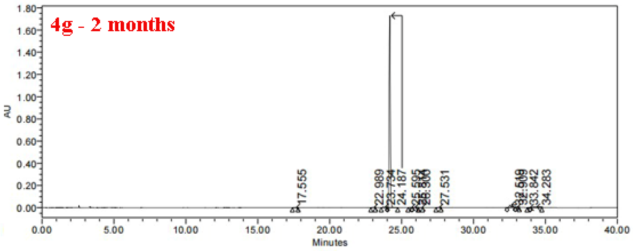
4g - 2 months
AU
Minutes

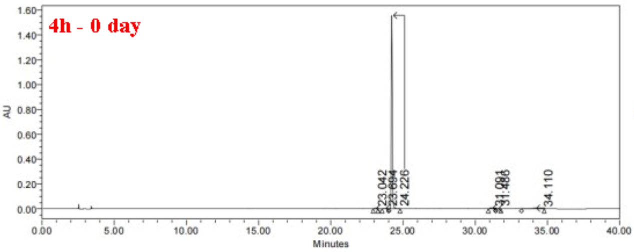
4h - 0 day
AU
Minutes

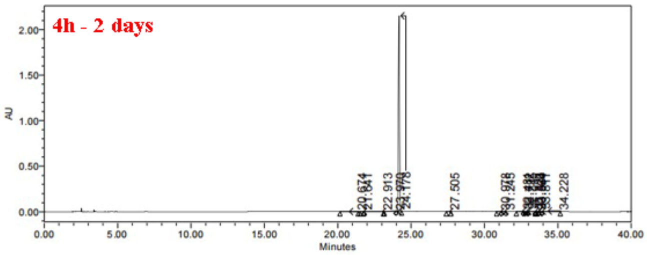
4h - 2 days
AU
Minutes

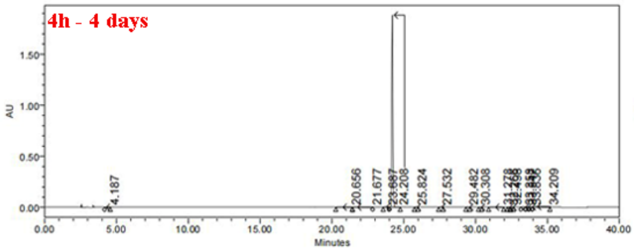
4h - 4 days
AU
Minutes

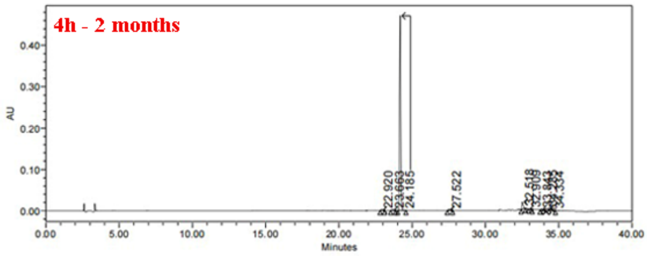
4h - 2 months
AU
Minutes

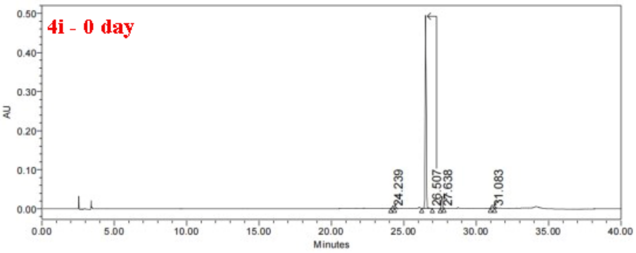
4i - 0 day
AU
Minutes

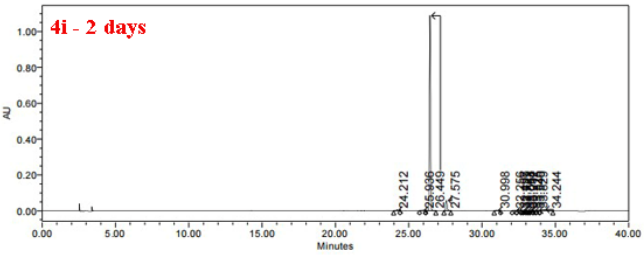
4i - 2 days
AU
Minutes

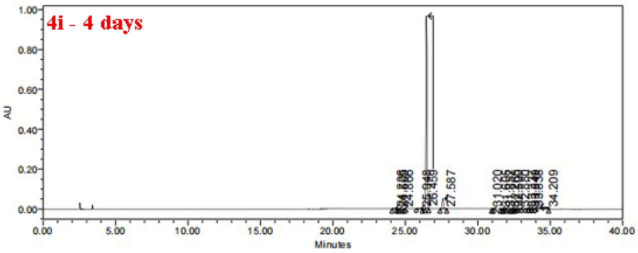
4i - 4 days
AU
Minutes

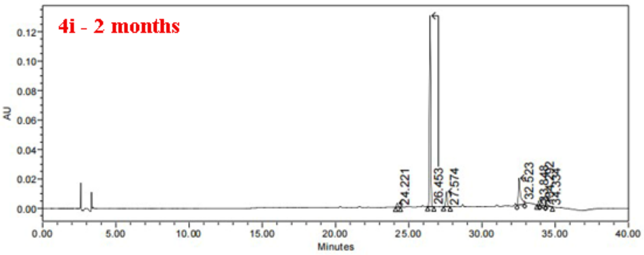
4i - 2 months
AU
Minutes

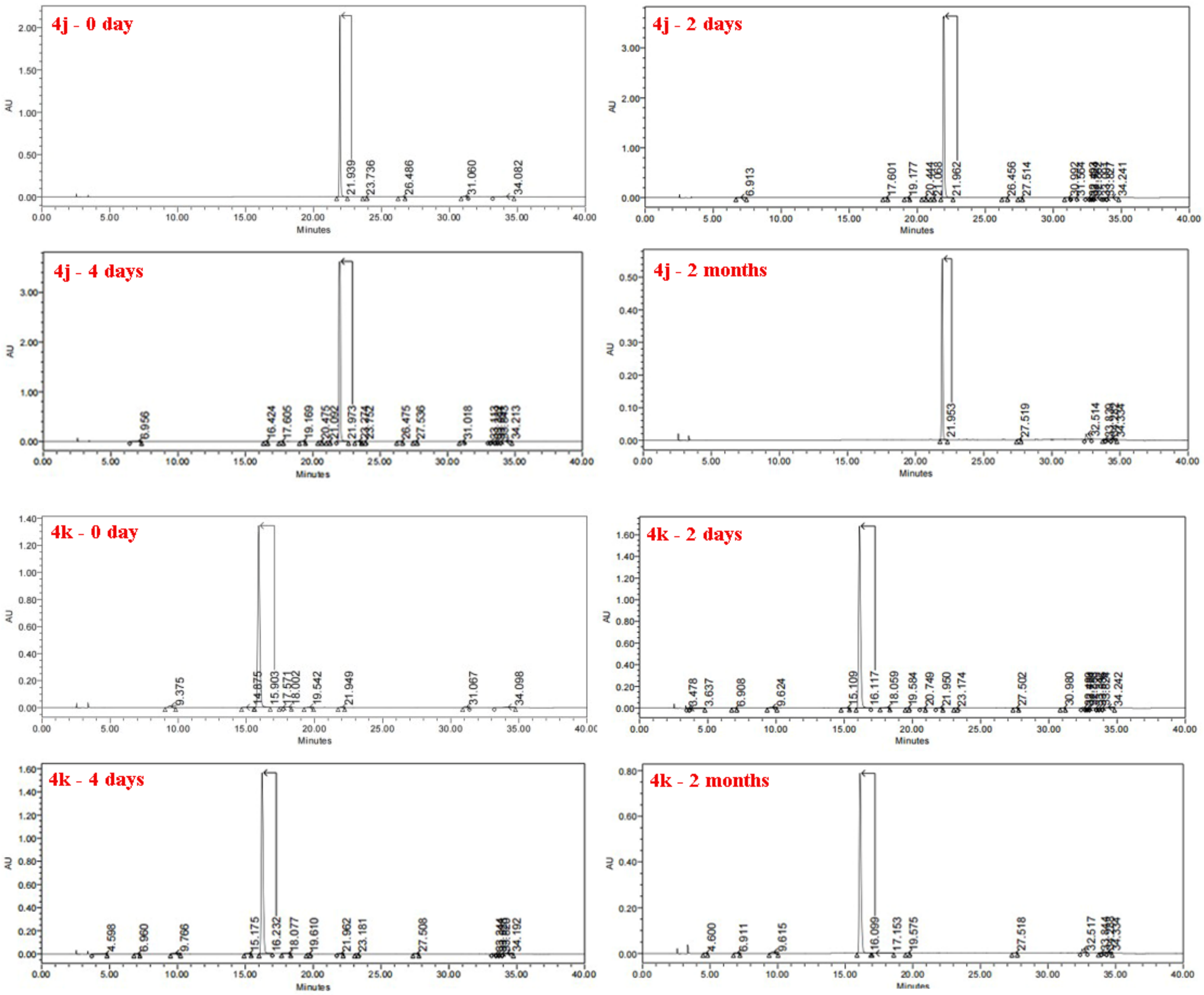
4j - 0 day
4j - 2 days
4j - 4 days
4j - 2 months
4k - 0 day
4k - 2 days
4k - 4 days
4k - 2 months
AU
Minutes

Table S3. ADMET properties of the compounds.

ADMET properties		Compound 4b	Kojic acid	Tropolone
**Absorption**				
Caco-2 permeability (cm/s) [a]		0.572	0.637	1.558
P-gp Ⅰ protein inhibitor [a]		No	No	No
P-gp Ⅱ protein inhibitor [a]		No	No	No
P-gp substrate [a]		Yes	No	No
Human intestinal absorption [a]		89.027%	93.152%	98.108%
**Distribution**				
Plasma protein binding[b]		97.0%	23.3%	47.9%
Volume distribution (L/kg) [a]		-0.11	-0.086	-0.045
Blood–brain barrier[c]		No	No	Yes
**Metabolism**				
CYP450	CYP1A2 inhibitor[c]	Yes	No	No
	CYP2C19 inhibitor[c]	No	No	No
	CYP2C9 inhibitor[c]	No	No	No
	CYP2D6 inhibitor[c]	No	No	No
	CYP3A4 inhibitor[c]	No	No	No
**Elimination**				
Clearance rate (mL/min/kg) [a]		-0.081	0.638	0.169
T1/2 (h)[b]		1.227	1.827	1.709
**Toxicity**				
Hepatotoxicity [a]		No	No	No
Ames toxicity [a]		No	No	No
Skin sensitization [a]		No	No	No
hERG inhibition [a]		No	No	No
LD_{50} of acute toxicity (mol/kg)[d]		3.1502	2.0673	2.4899

LD_{50}: lethal dose; CYP: cytochrome p450; $T_{1/2}$: time required for the plasma concentration of a drug to decrease by 50%; hERG: human Ether-a-go-go-Related Gene; a: pkCSM; b: ADMETlab 3.0; c: SwissADME; d: admetSAR 2.0.

Table S4. Drug-likeness properties of the compounds.

Compound	MW[a] (g/mol)	HBA[a]	HBD[a]	RB[a]	TPSA[a] (Å²)	Log *P*[a]	MR[b]	RO5[b]	Ghose Filter[b]	Veber rule[b]	Egan rule[b]	Drug likeness
4a	218	4	4	3	66.2	2.101	65.06	Yes	Yes	Yes	Yes	Yes
4b	236	4	4	3	66.2	2.297	65.01	Yes	Yes	Yes	Yes	Yes
4c	252	4	4	3	66.2	3.001	70.07	Yes	Yes	Yes	Yes	Yes
4d	295	4	4	3	66.2	3.031	72.76	Yes	Yes	Yes	Yes	Yes
4e	248	5	4	4	75.43	1.901	71.55	Yes	Yes	Yes	Yes	Yes
4f	246	4	3	4	55.34	2.445	74.77	Yes	Yes	Yes	Yes	Yes
4g	232	4	3	3	55.34	2.318	69.96	Yes	Yes	Yes	Yes	Yes
4h	262	5	3	4	64.57	2.223	76.45	Yes	Yes	Yes	Yes	Yes
4i	309	4	3	3	55.34	3.093	77.66	Yes	Yes	Yes	Yes	Yes
4j	276	6	4	5	92.5	2.108	76.34	Yes	Yes	Yes	Yes	Yes
4k	234	5	5	3	86.43	1.39	67.08	Yes	Yes	Yes	Yes	Yes
Kojic acid	142	4	2	1	70.67	-0.56	33.13	Yes	No*	Yes	Yes	No
Tropolone	122	2	1	0	37.3	0.667	34.74	Yes	No*	Yes	Yes	No

MW: molecular weight; HBA: number of H-Bond acceptors; HBD: number of H-Bond donors; RB: number of rotatable bonds; TPSA: Topological Polar Surface Area; MR: molar refractivity; a: ADMETlab 3.0; b: SwissADME; *: three violations: MW < 160, MR < 40, and atoms < 20.

Table S5. Two-dimensional molecular docking plots of the synthesized compounds with mTYR and TYRP1.

mTYR:

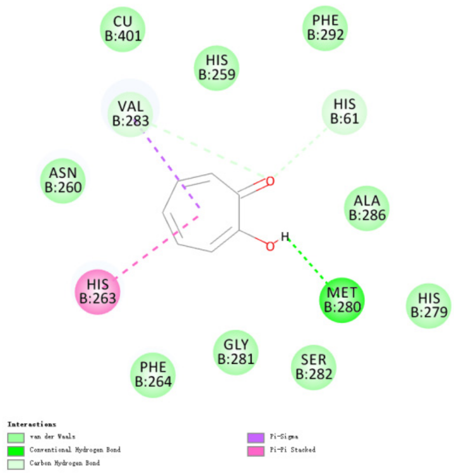

Tropolone

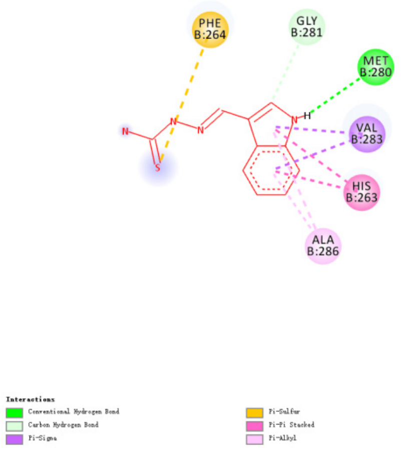

4a

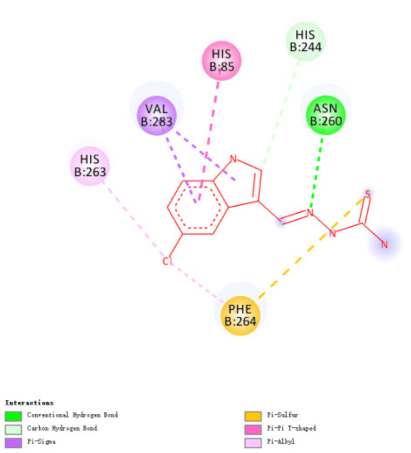

4c

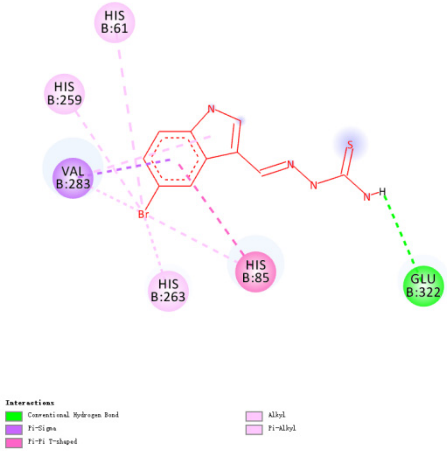

4d

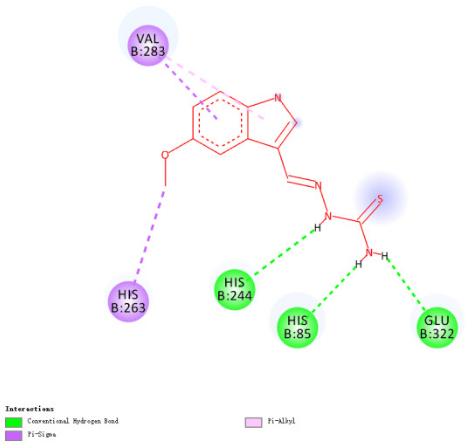

4e

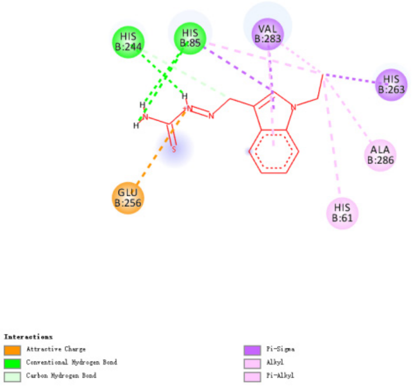

4f

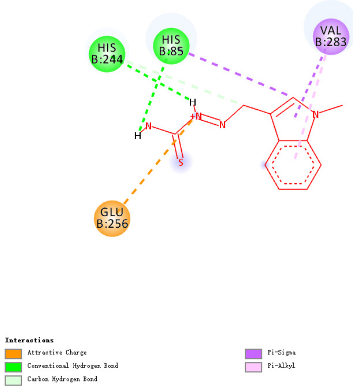

4g

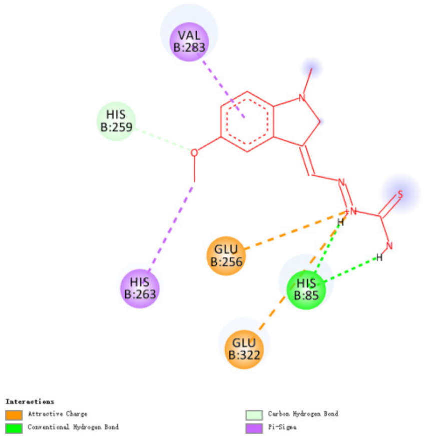

4h

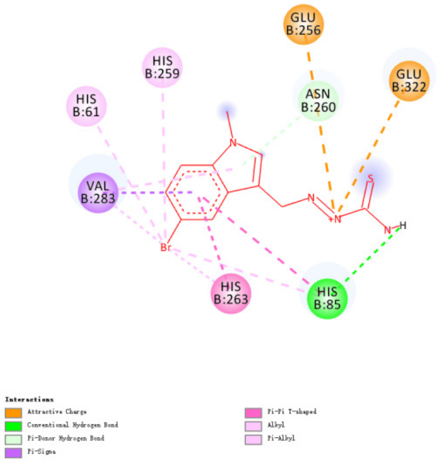

4i

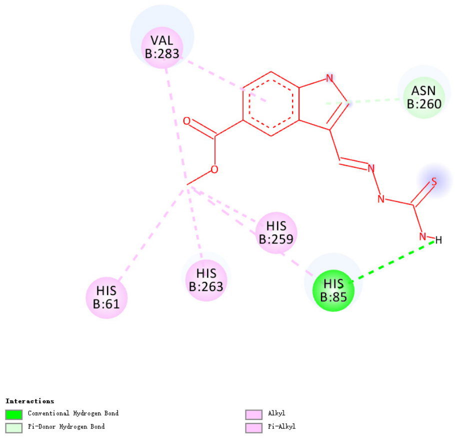

4j

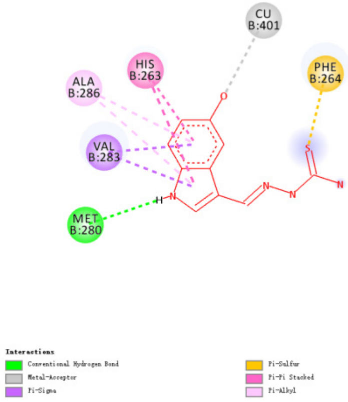

4k

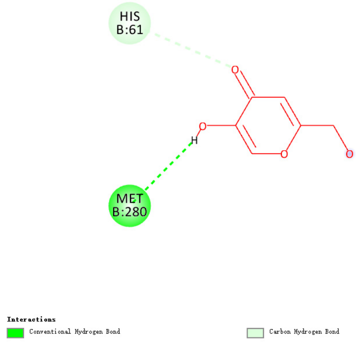

Kojic acid

TYRP1:

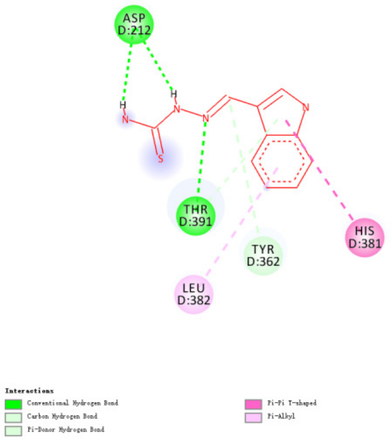

4a

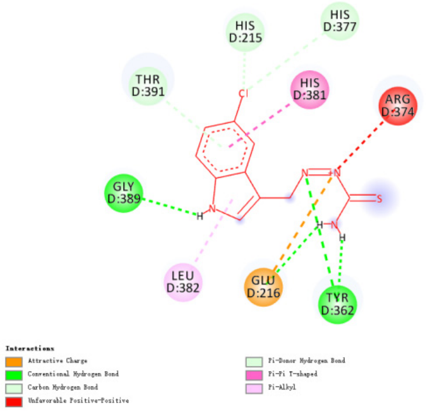

4c

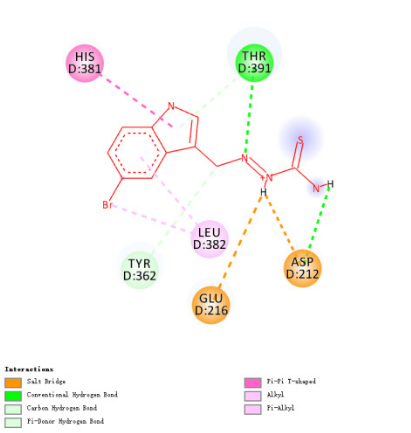

4d

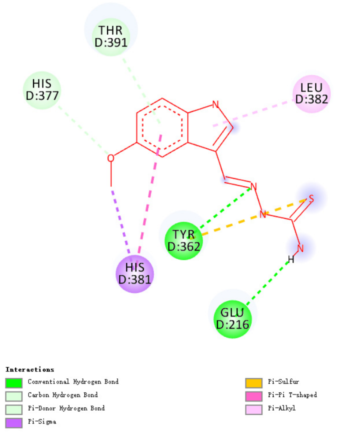

4e

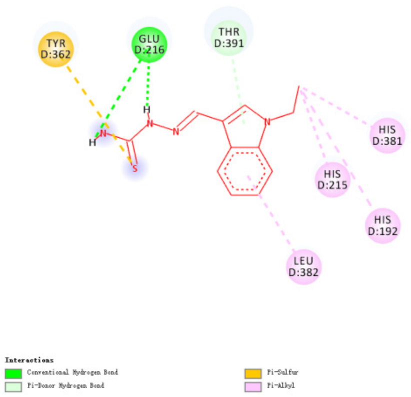

4f

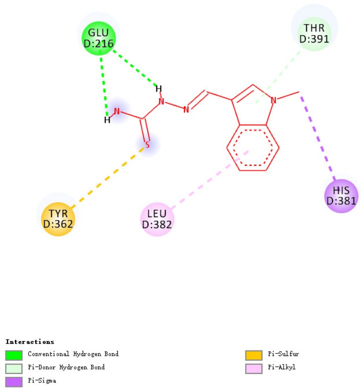

4g

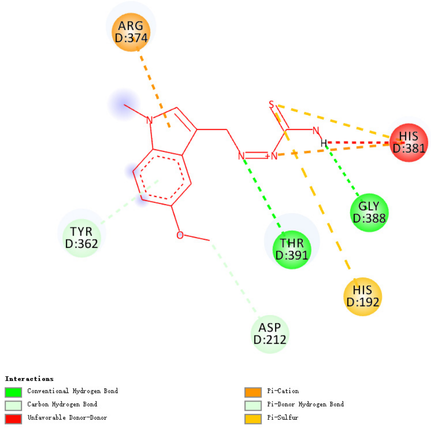

4h

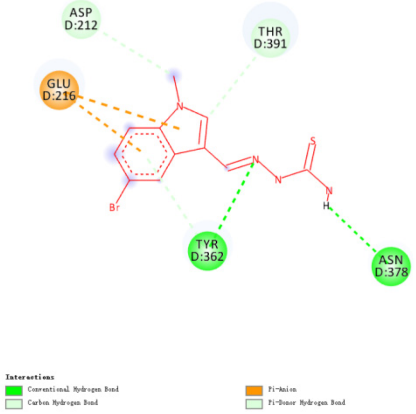

4i

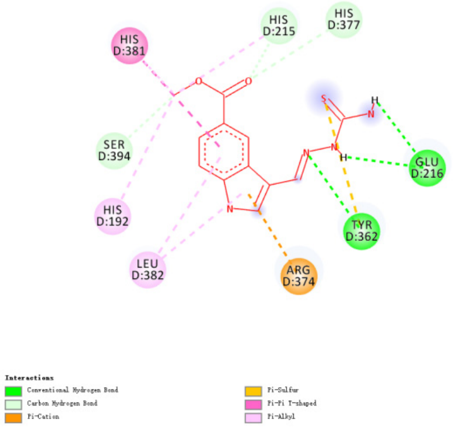

4j

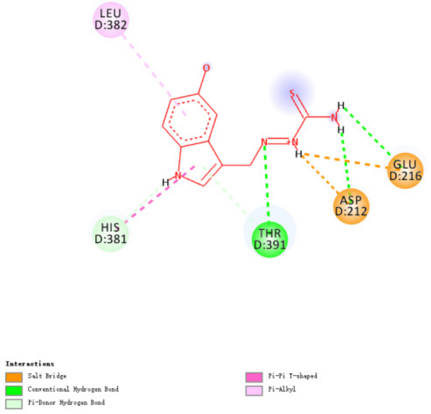

4k

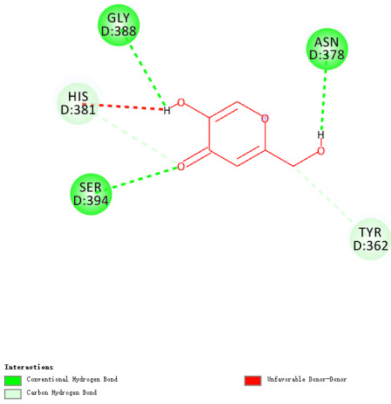

Kojic acid

Table S6. The docking energy of ten repeated molecular dockings of compound **4b** with tyrosinase.

No.	Binding energy (kcal/mol)	
	mTYR	TYRP1
1	-7.0	-6.5
2	-7.0	-6.5
3	-7.0	-6.6
4	-7.0	-6.5
5	-6.9	-6.5
6	-7.0	-6.5
7	-7.0	-6.4
8	-7.0	-6.5
9	-7.0	-6.5
10	-7.0	-6.5

Figure S1. The inhibition percentage of DPPH by the synthesized compound.

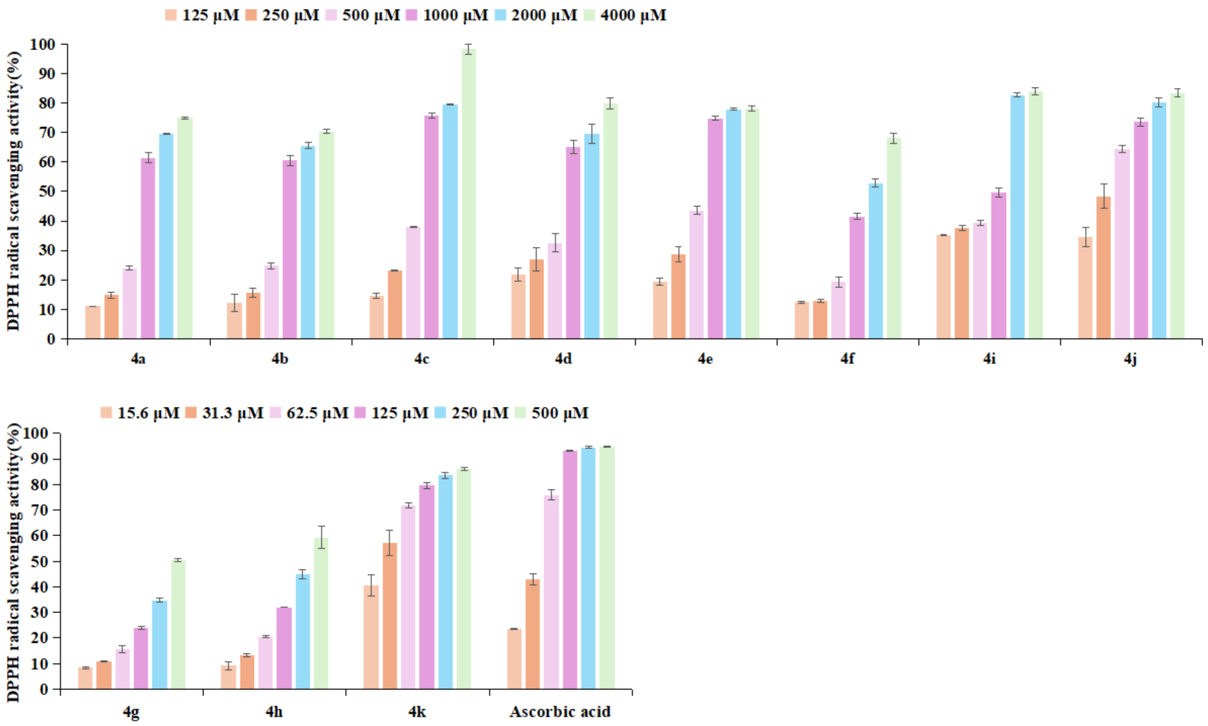

Figure S2. ^{1}H NMR of the 1-ethyl-1H-indole-3-carbaldehyde compound (**2f**).

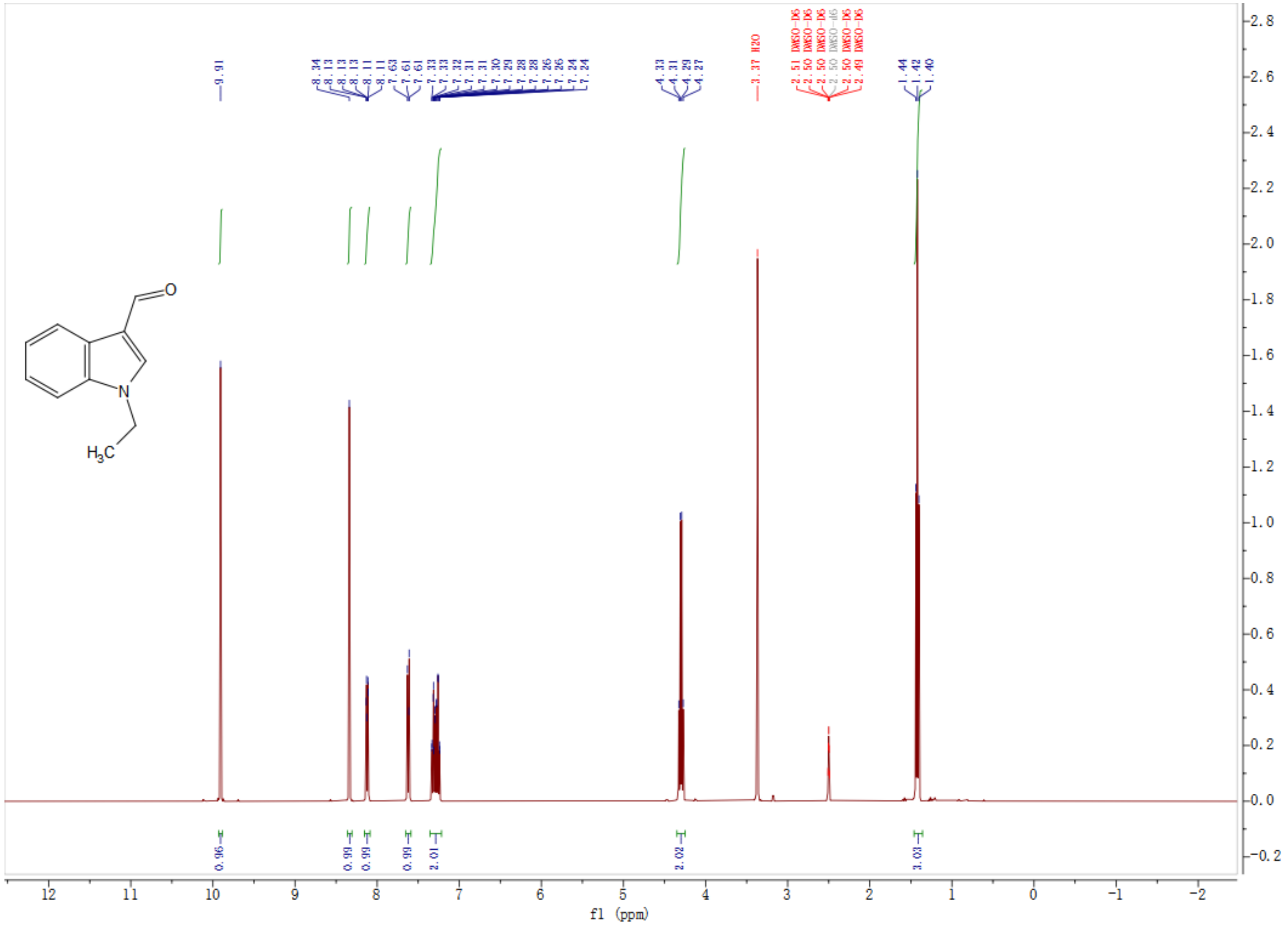

Figure S3. ^{1}H NMR of the 1-methyl-1H-indole-3-carbaldehyde compound (**2g**).

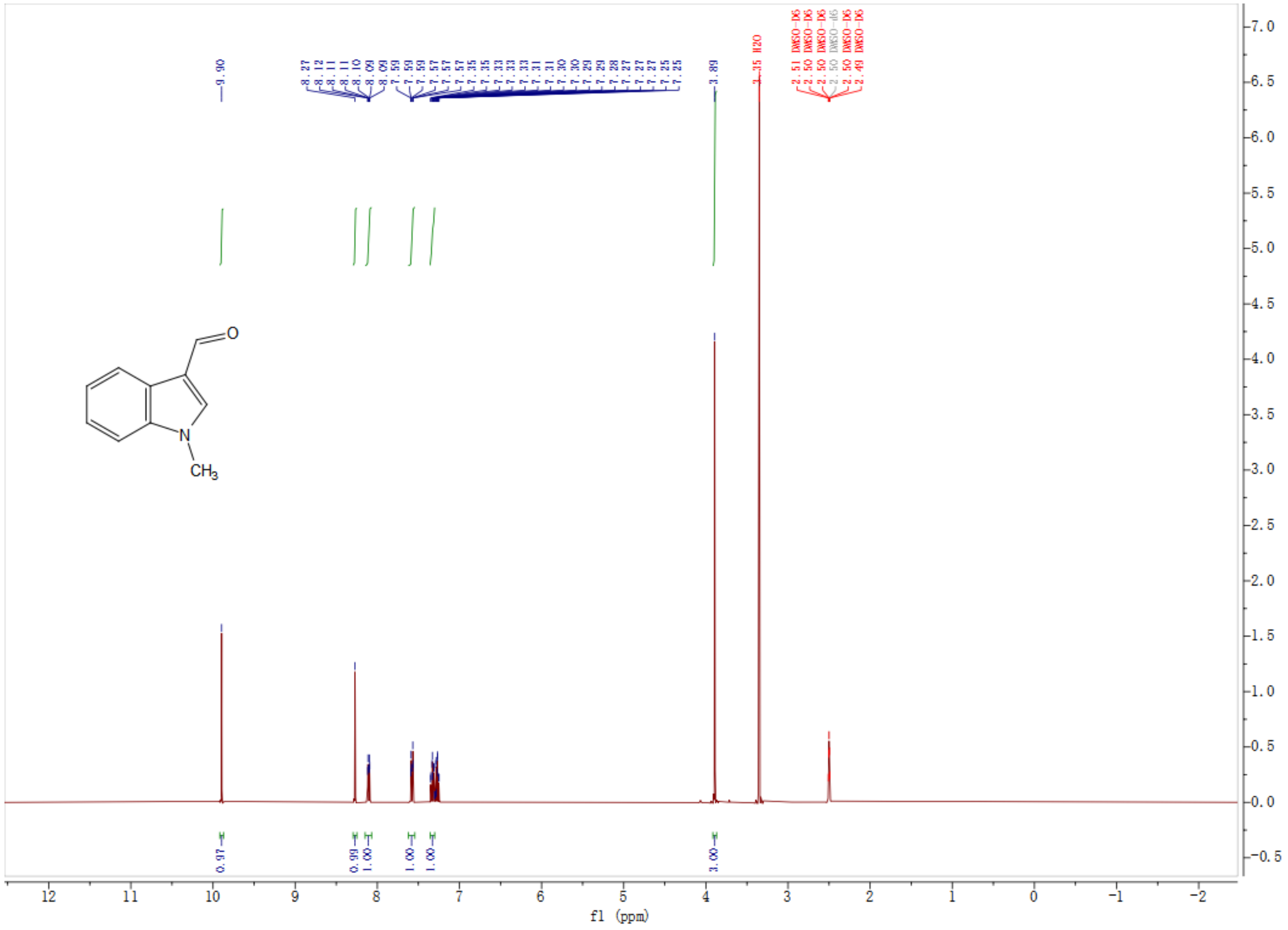

Figure S4. ^{1}H NMR of the (E)-2-((1H-indol-3-yl)methylene)hydrazine-1-carbothioamide compound (**4a**).

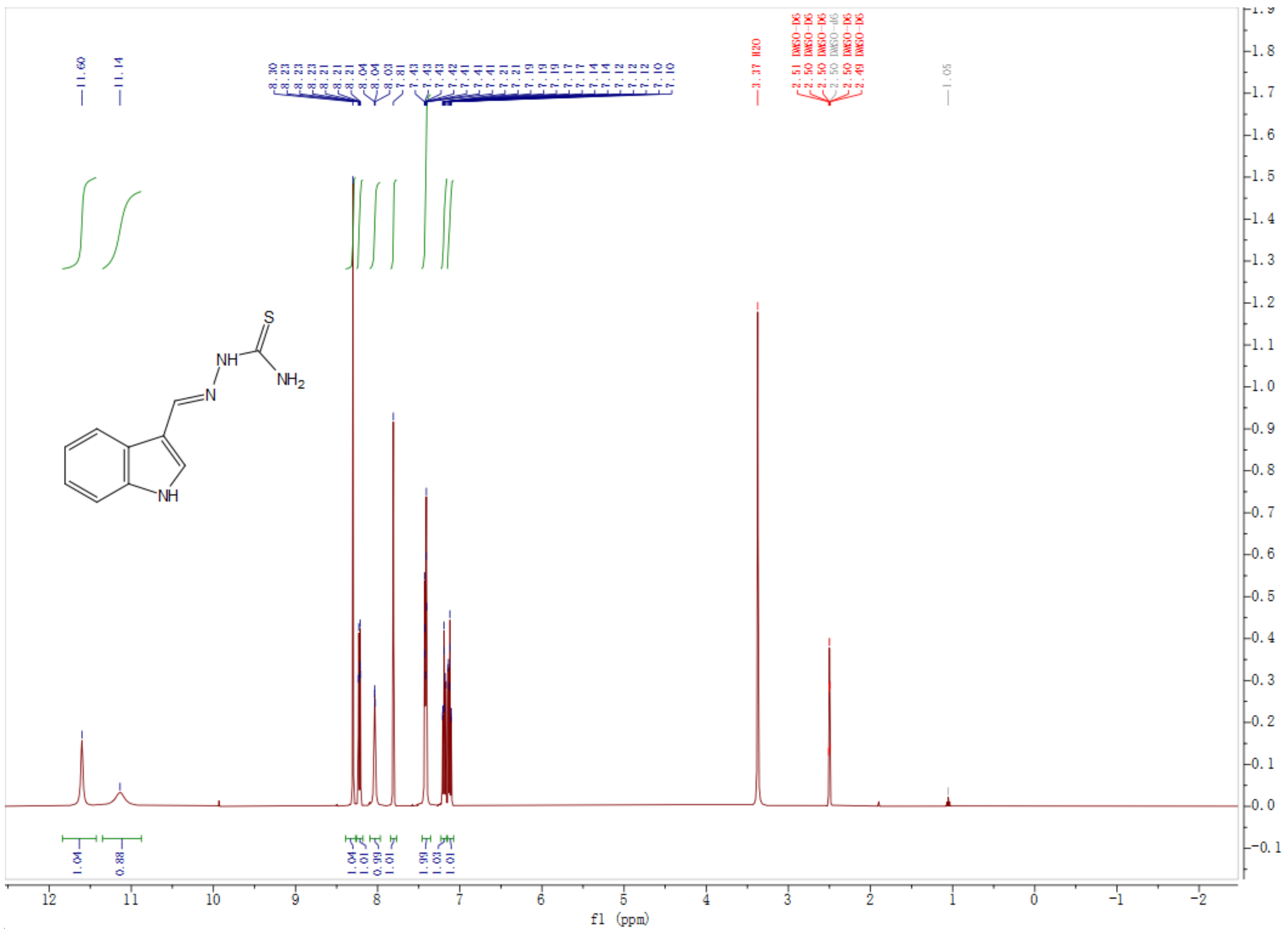

Figure S5. HRMS of the (E)-2-((1H-indol-3-yl)methylene)hydrazine-1-carbothioamide compound (**4a**).

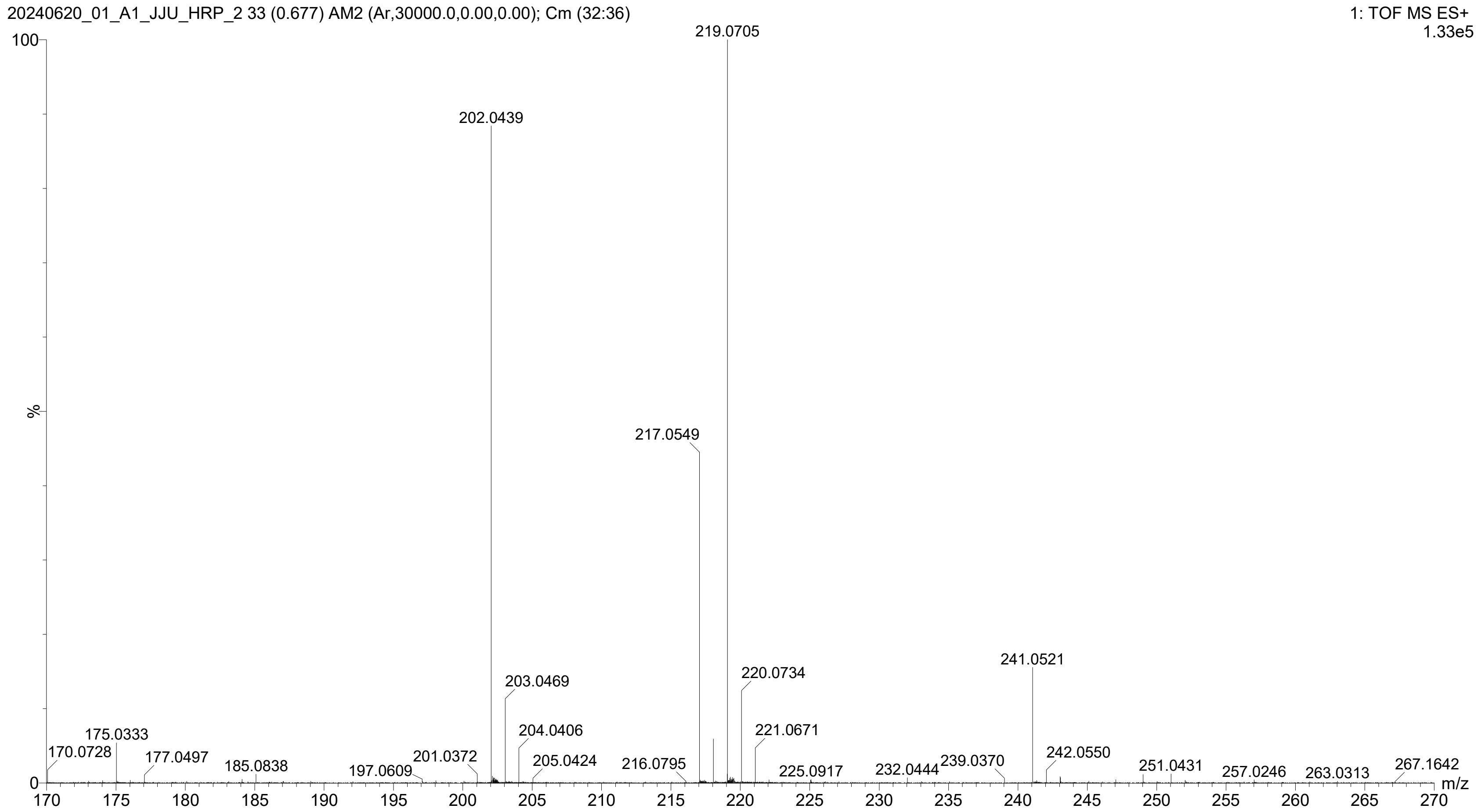

Figure S6. ^{1}H NMR of the (E)-2-((5-fluoro-1H-indol-3-yl)methylene)hydrazine-1-carbothioamide compound (**4b**).

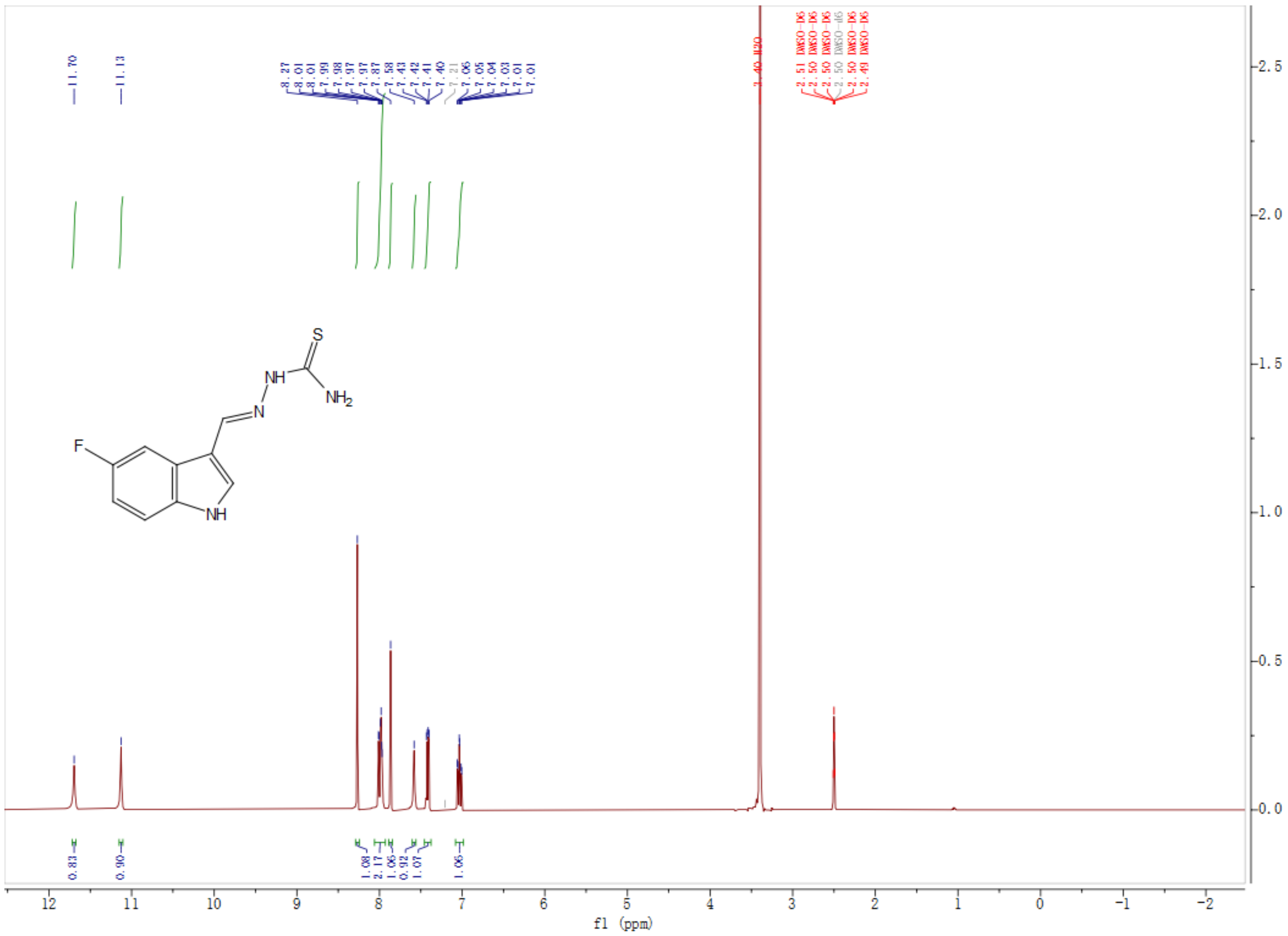

Figure S7. ^{13}C NMR of the (E)-2-((5-fluoro-1H-indol-3-yl)methylene)hydrazine-1-carbothioamide compound (**4b**).

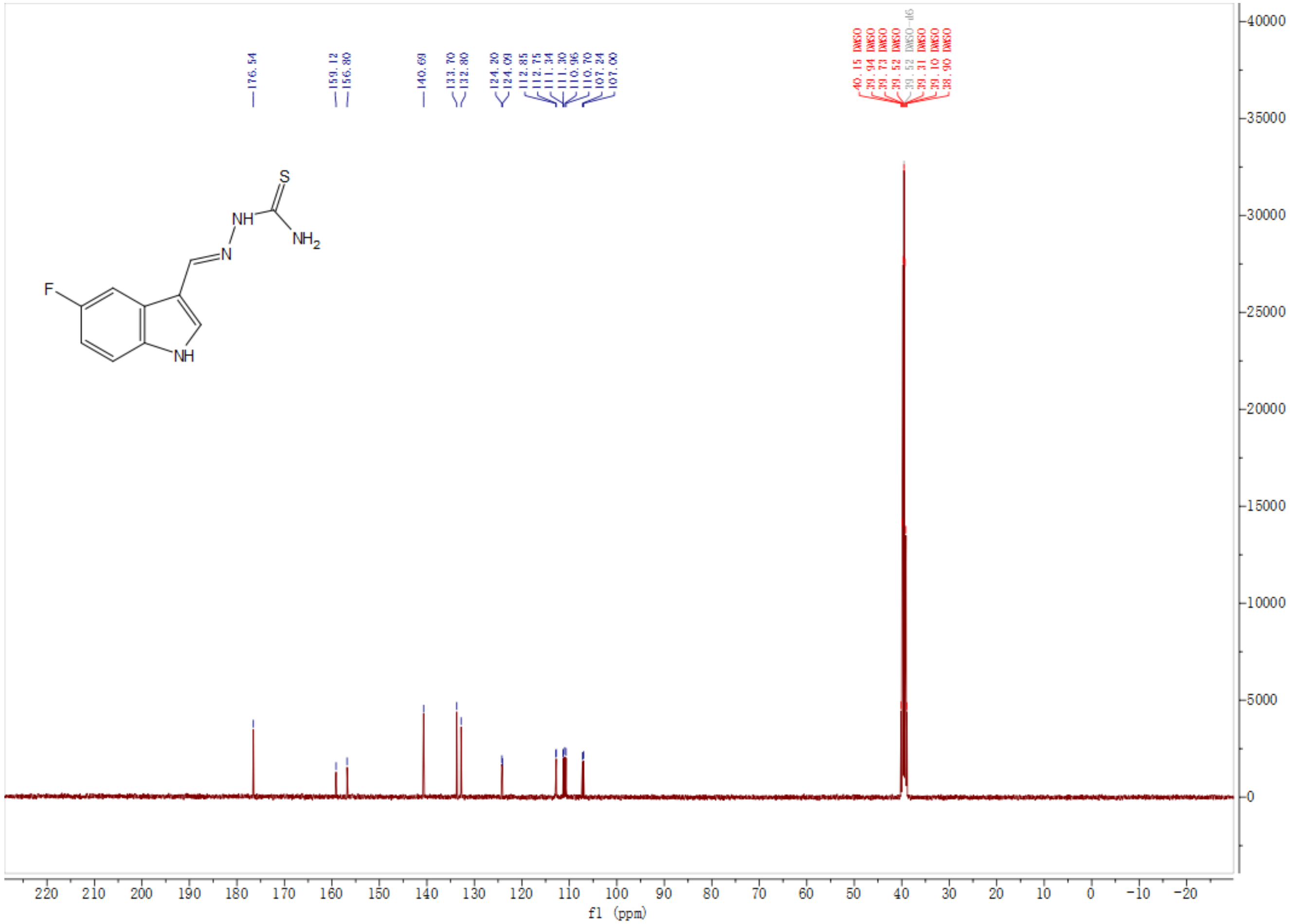

Figure S8. HRMS of the (E)-2-((5-fluoro-1H-indol-3-yl)methylene)hydrazine-1-carbothioamide compound (**4b**).

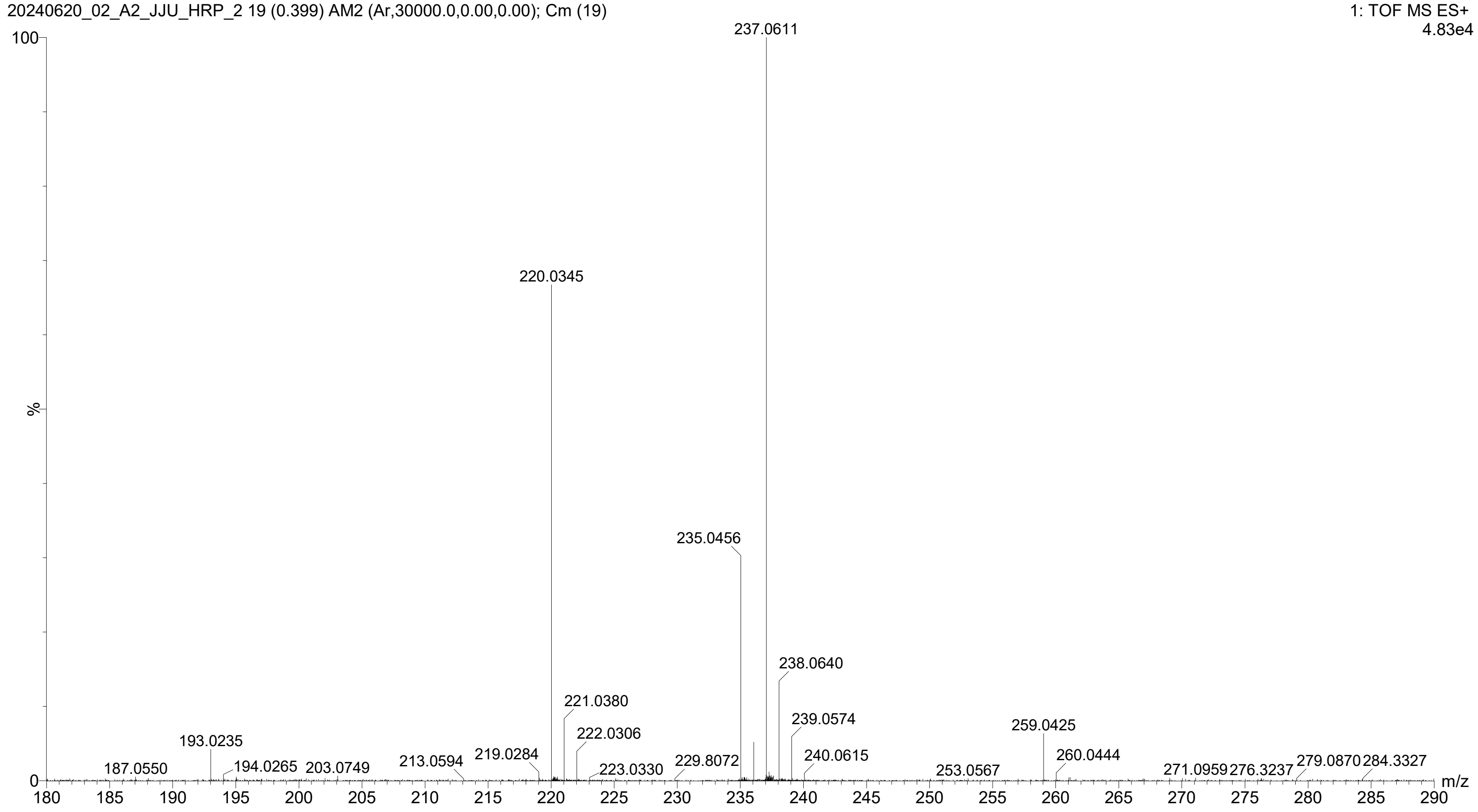

Figure S9. ^{1}H NMR of the (E)-2-((5-chloro-1H-indol-3-yl)methylene)hydrazine-1-carbothioamide compound (**4c**).

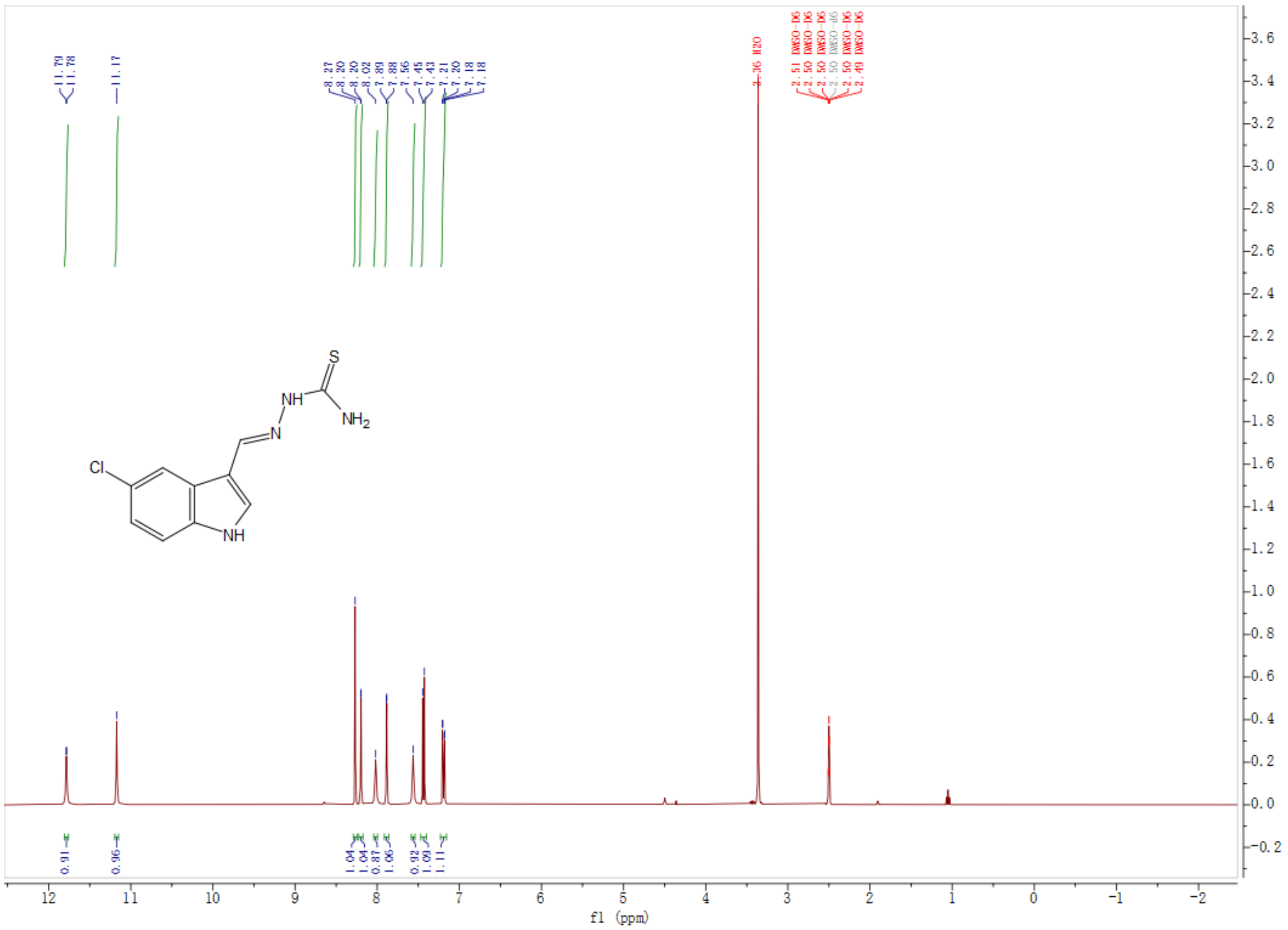

Figure S10. ^{13}C NMR of the (E)-2-((5-chloro-1H-indol-3-yl)methylene)hydrazine-1-carbothioamide compound (**4c**).

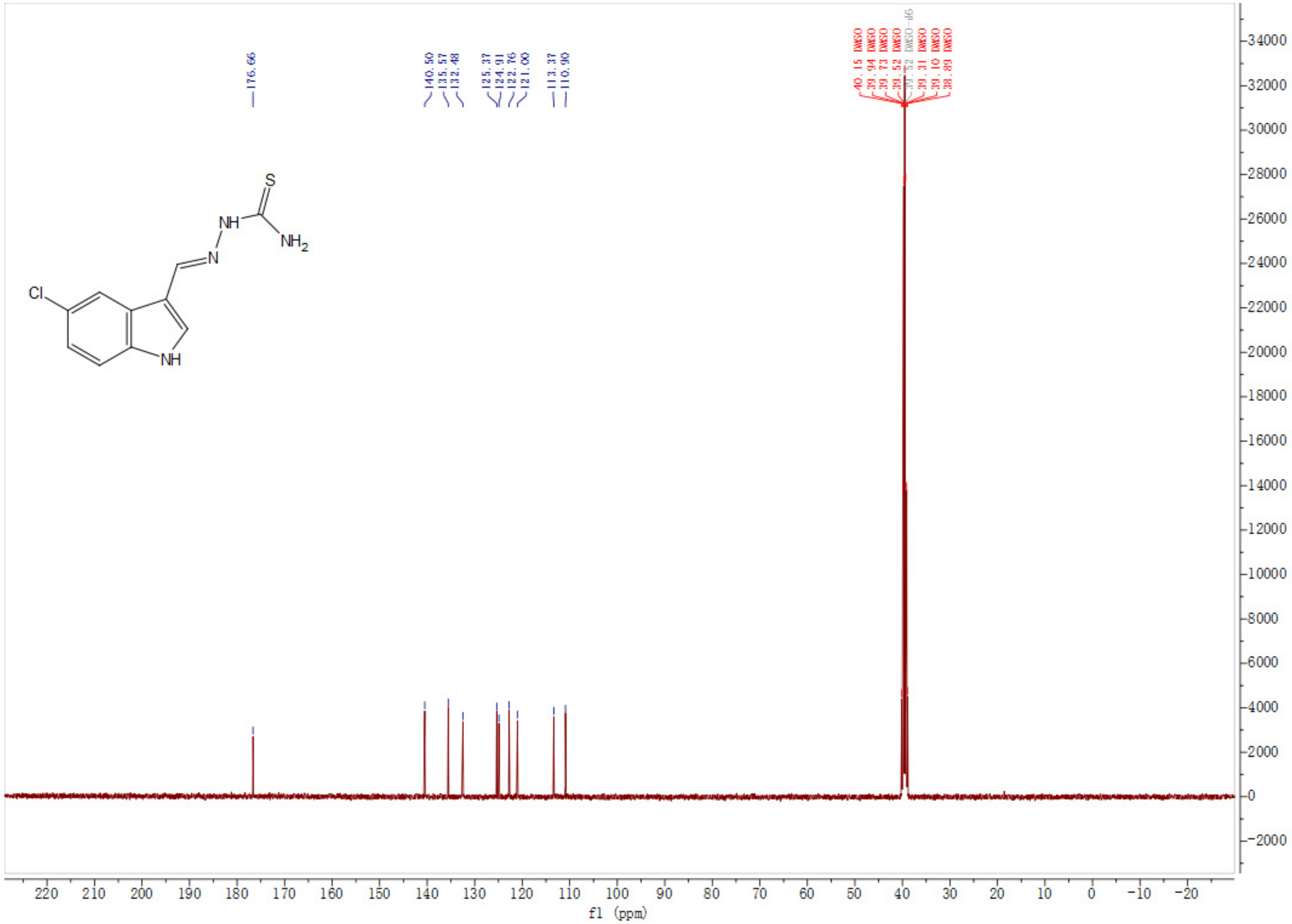

Figure S11. HRMS of the (E)-2-((5-chloro-1H-indol-3-yl)methylene)hydrazine-1-carbothioamide compound (**4c**).

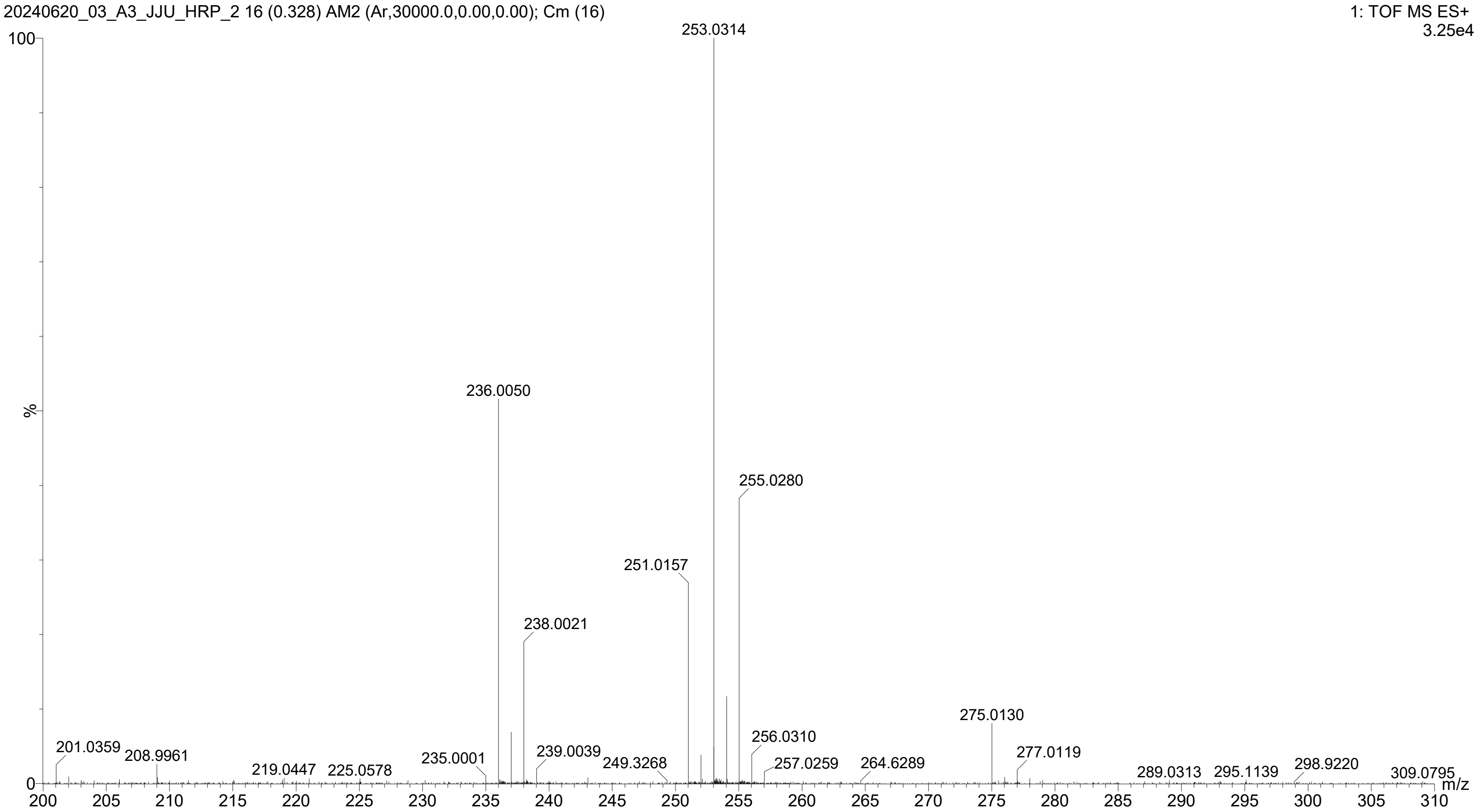

Figure S12. ^{1}H NMR of the (E)-2-((5-bromo-1H-indol-3-yl)methylene)hydrazine-1-carbothioamide compound (**4d**).

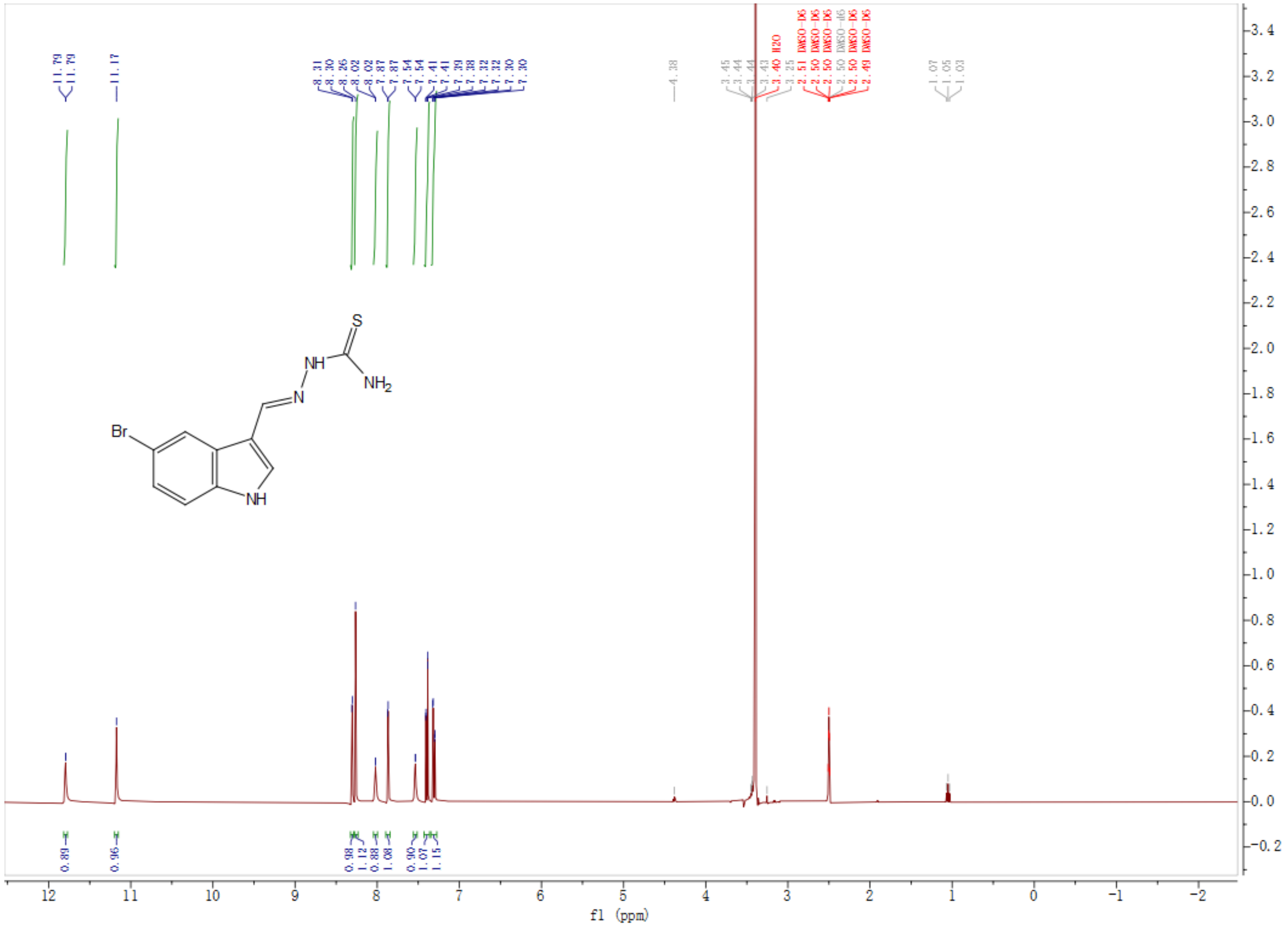

Figure S13. HRMS of the (E)-2-((5-bromo-1H-indol-3-yl)methylene)hydrazine-1-carbothioamide compound (**4d**).

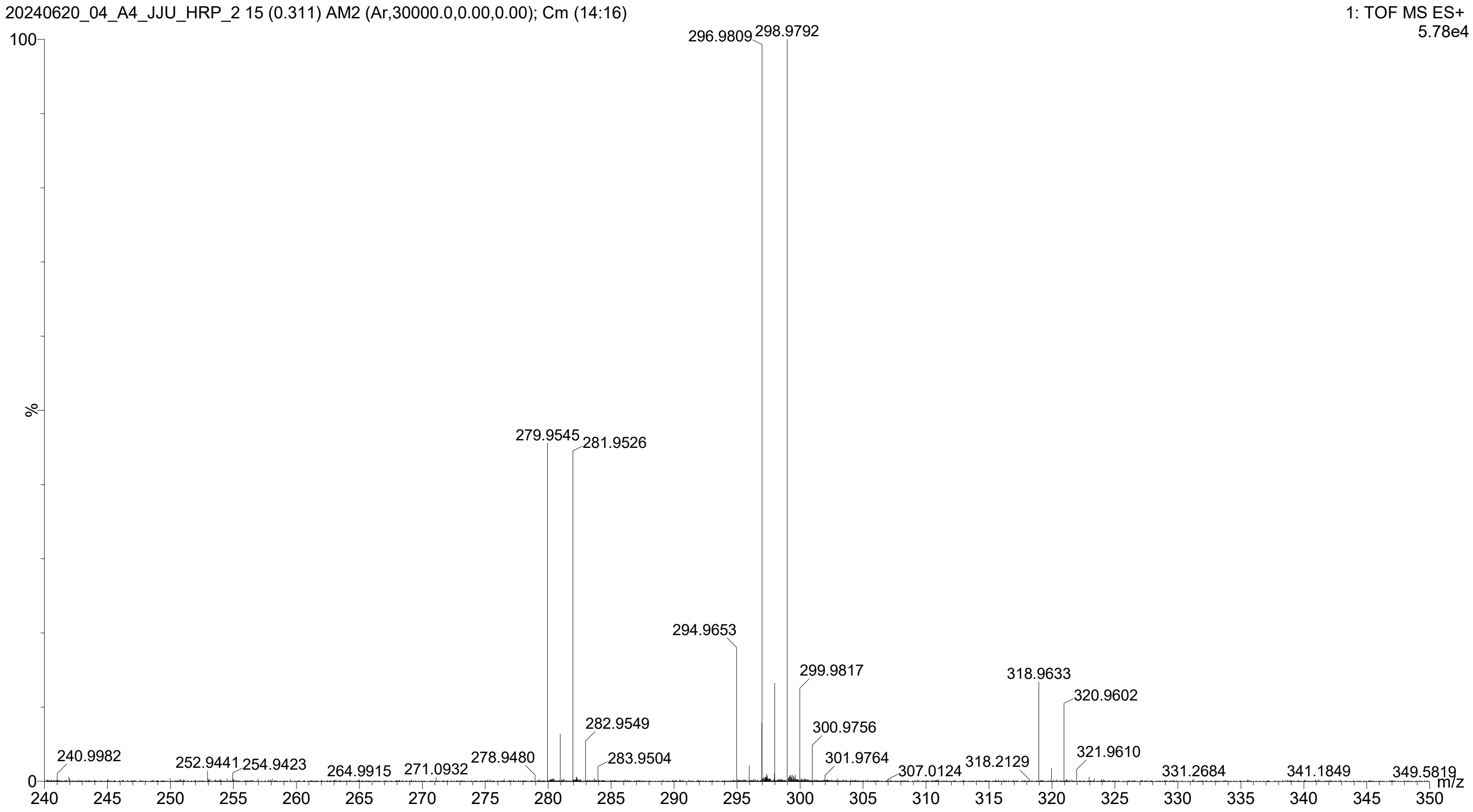

Figure S14. ^{1}H NMR of the (E)-2-((5-methoxy-1H-indol-3-yl)methylene)hydrazine-1-carbothioamide compound (**4e**).

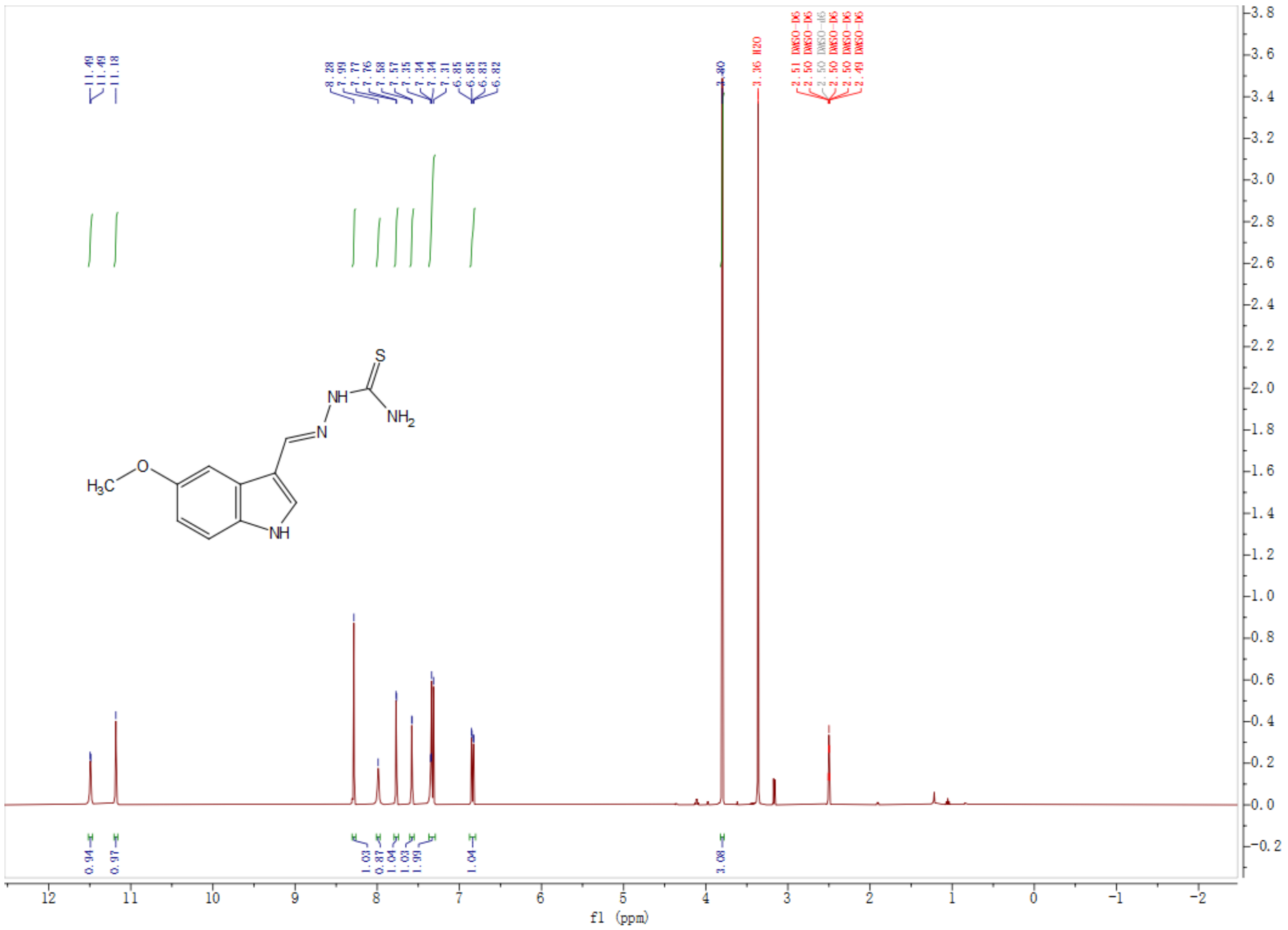

Figure S15. ^{13}C NMR of the (E)-2-((5-methoxy-1H-indol-3-yl)methylene)hydrazine-1-carbothioamide compound **(4e)**.

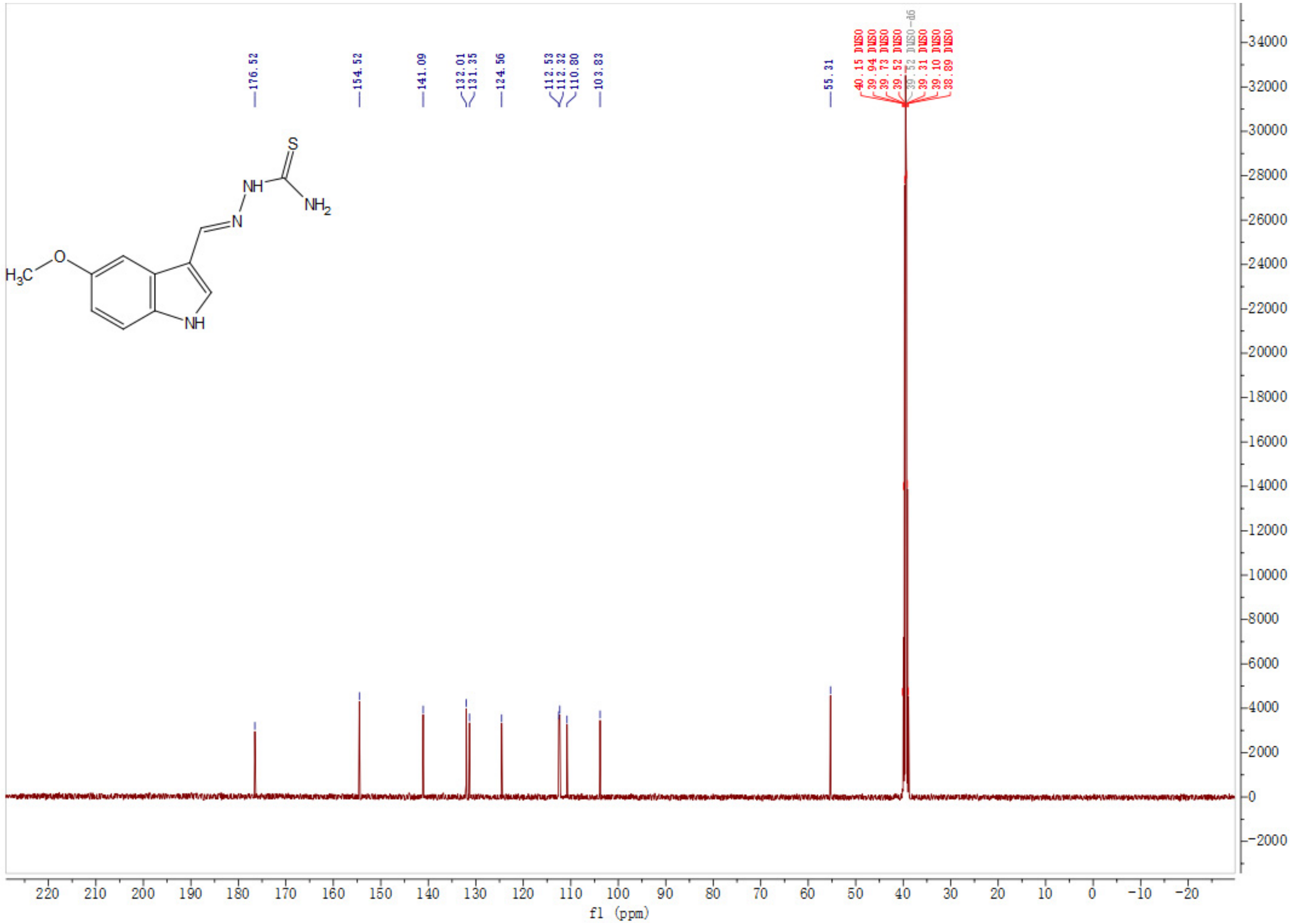

Figure S16. HRMS of the (E)-2-((5-methoxy-1H-indol-3-yl)methylene)hydrazine-1-carbothioamide compound (**4e**).

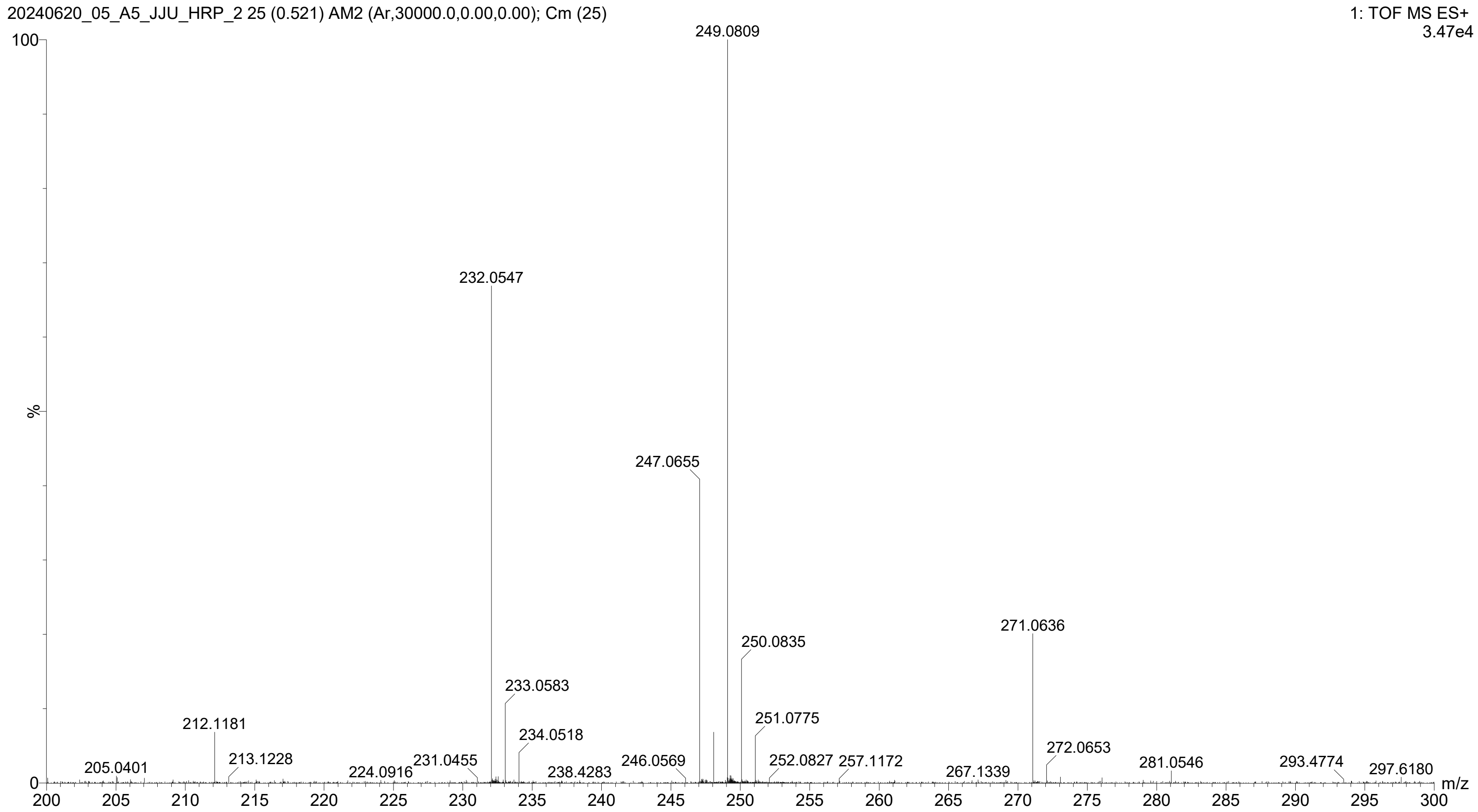

Figure S17. ^{1}H NMR of the (E)-2-((1-ethyl-1H-indol-3-yl)methylene)hydrazine-1-carbothioamide compound (**4f**).

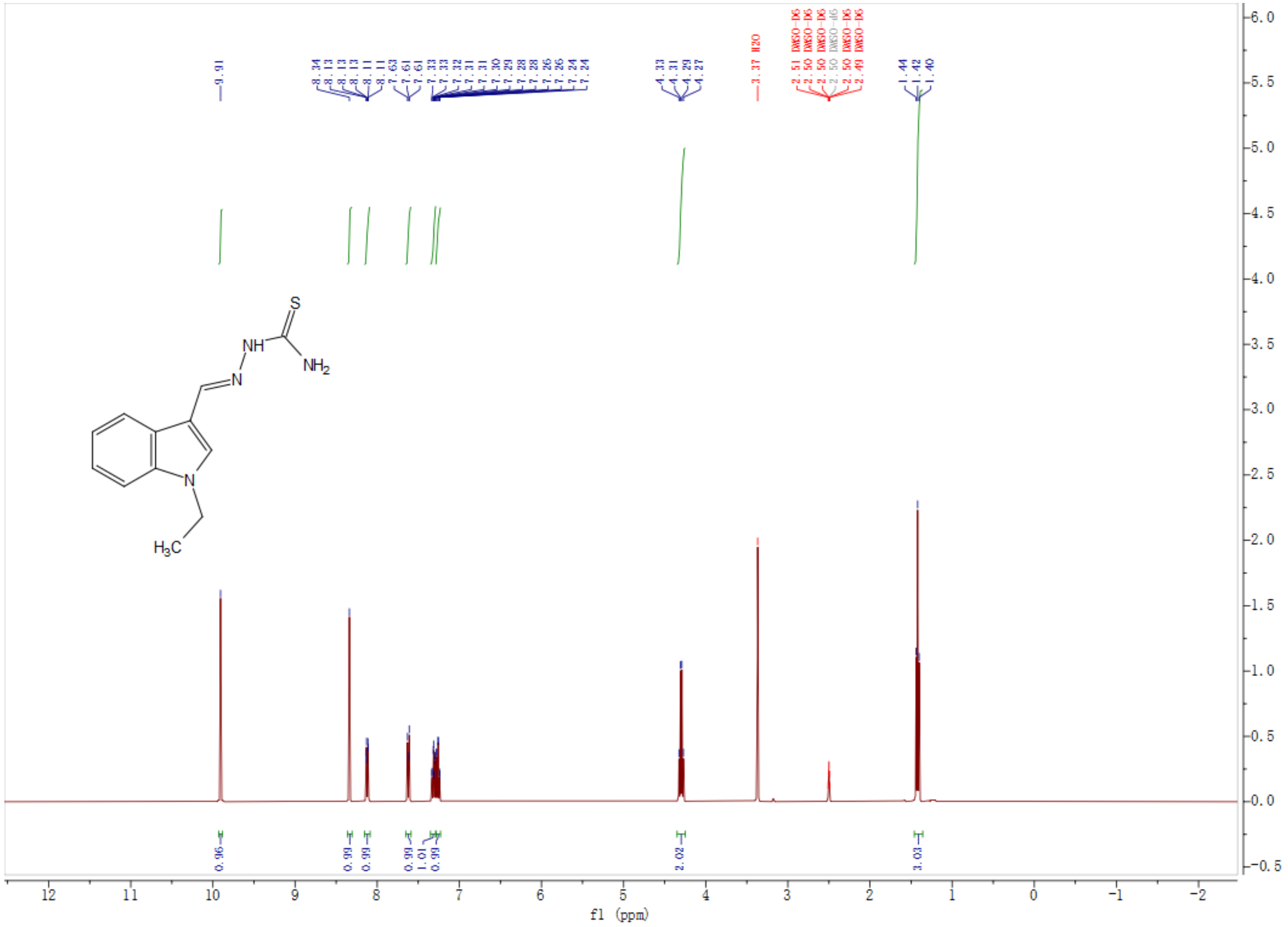

Figure S18. ^{13}C NMR of the (E)-2-((1-ethyl-1H-indol-3-yl)methylene)hydrazine-1-carbothioamide compound (**4f**).

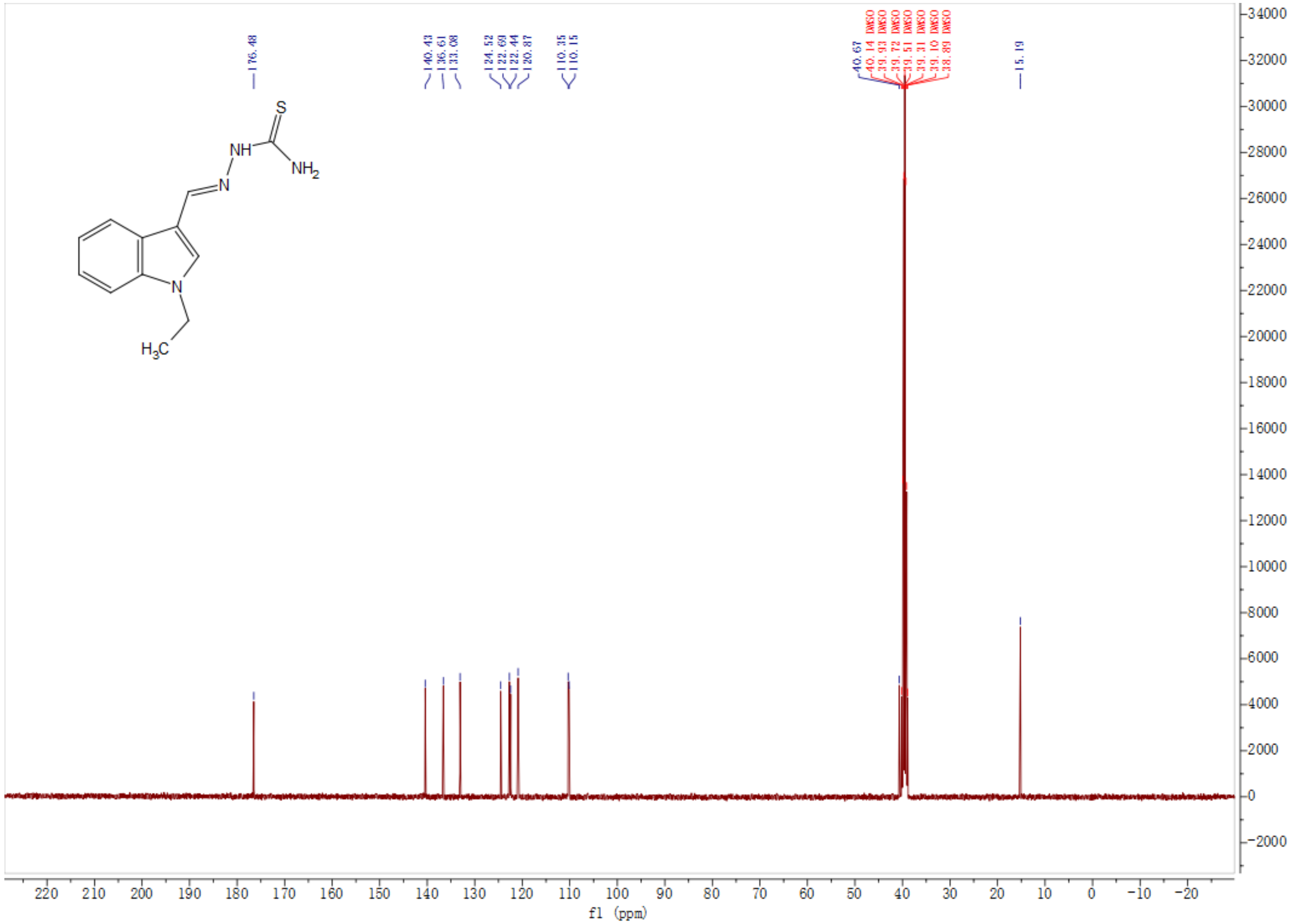

Figure S19. HRMS of the (E)-2-((1-ethyl-1H-indol-3-yl)methylene)hydrazine-1-carbothioamide compound (**4f**).

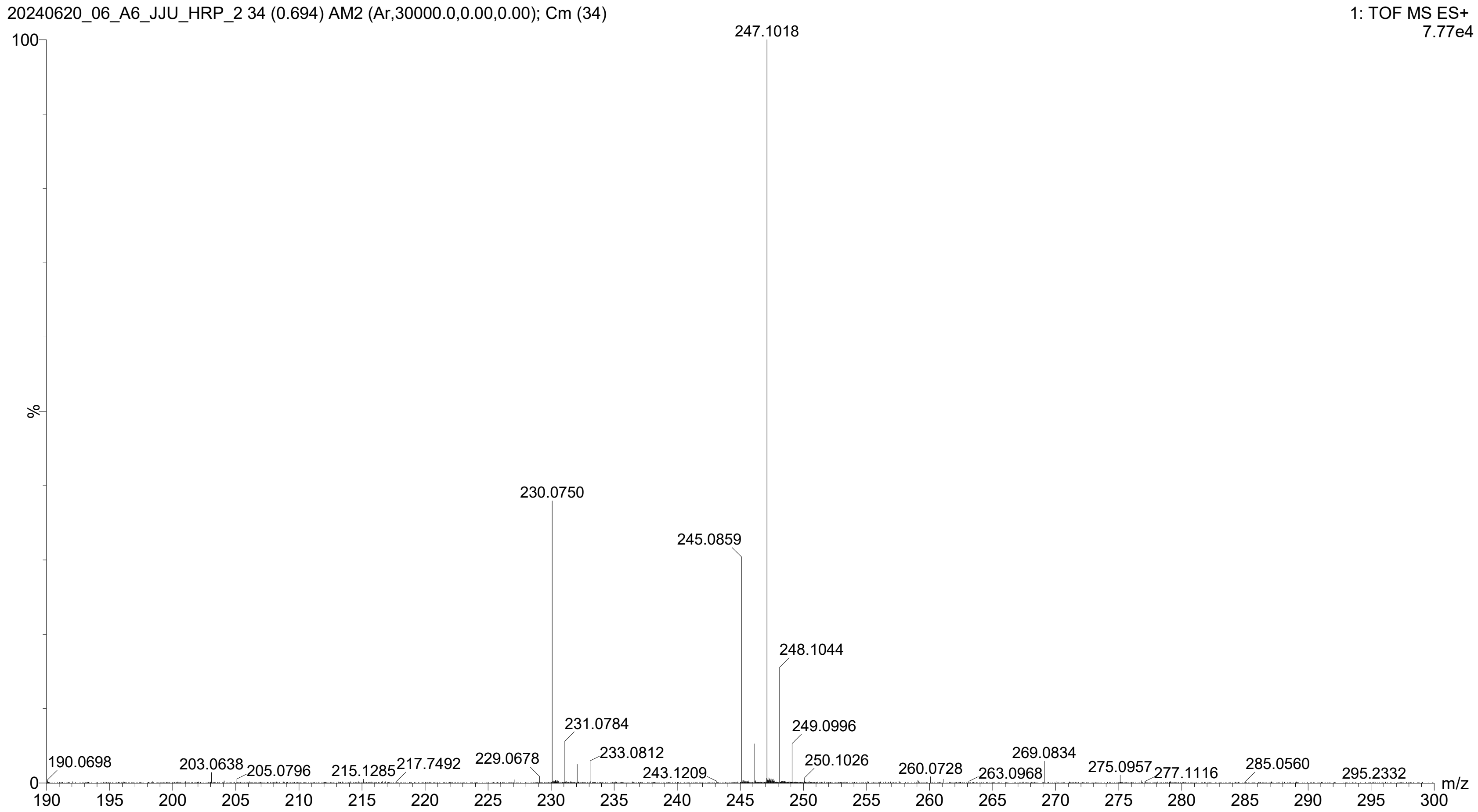

Figure S20. ¹H NMR of the (E)-2-((1-methyl-1H-indol-3-yl)methylene)hydrazine-1-carbothioamide compound (**4g**).

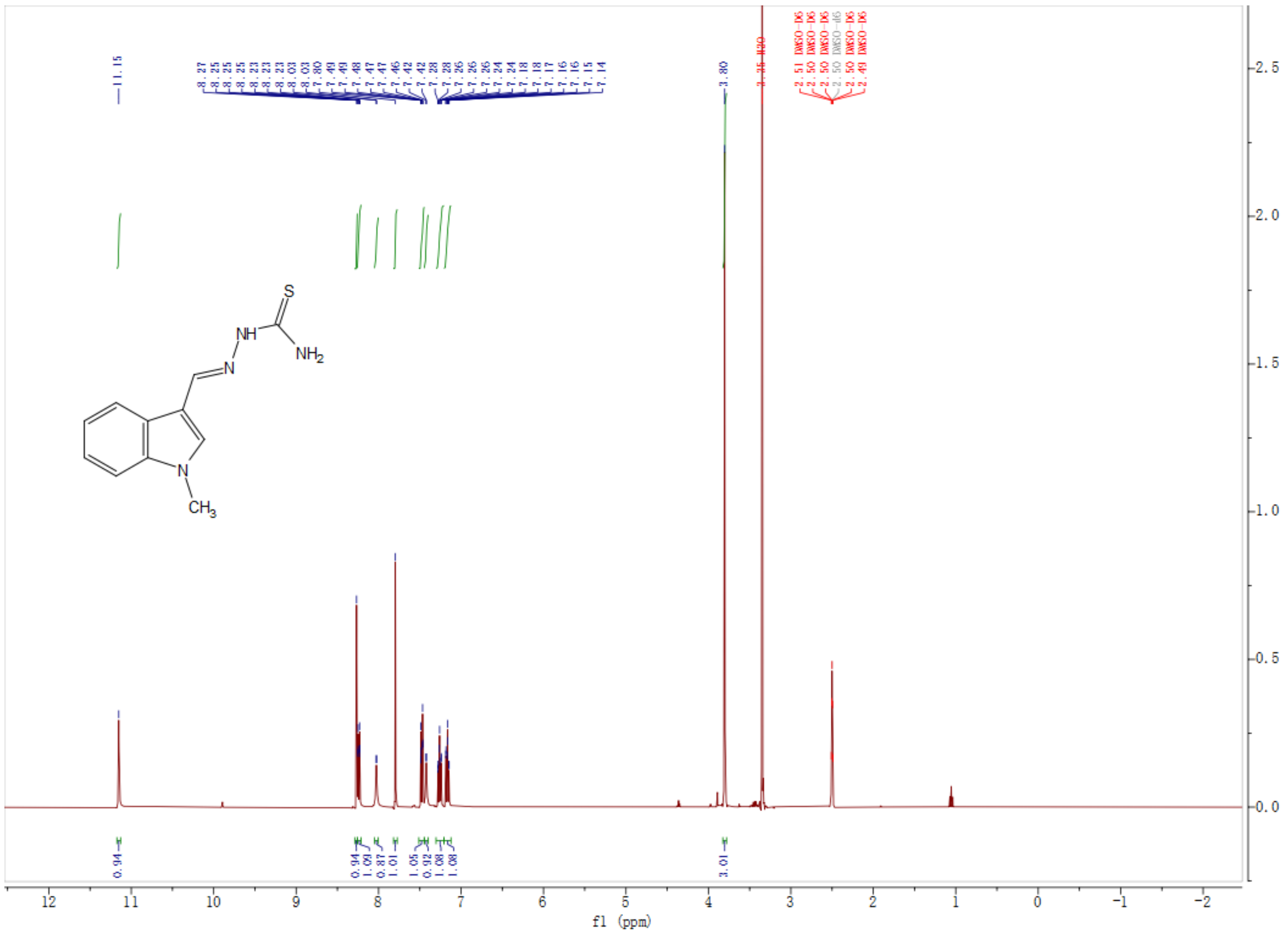

Figure S21. HRMS of the (E)-2-((1-methyl-1H-indol-3-yl)methylene)hydrazine-1-carbothioamide compound (**4g**).

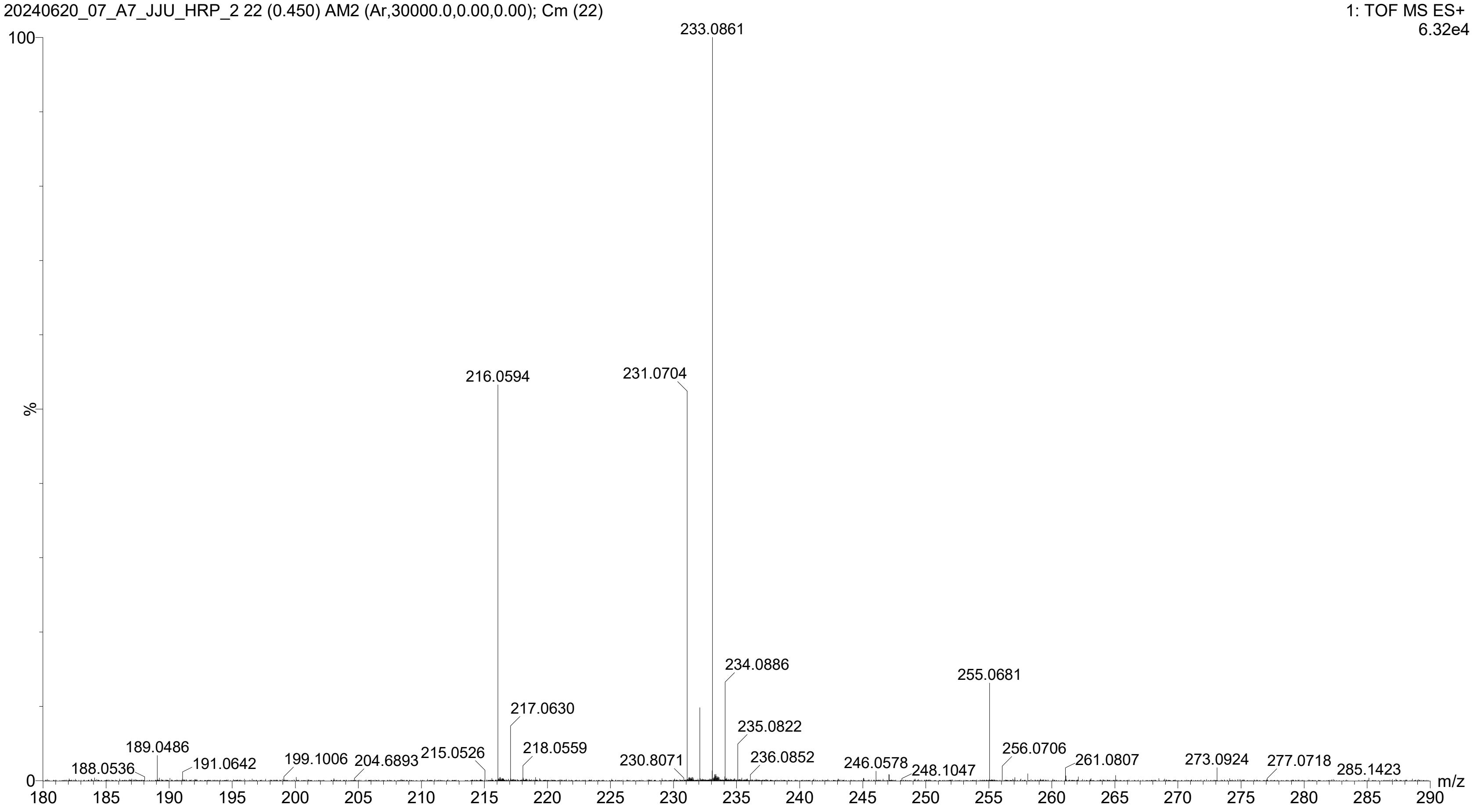

Figure S22. ^{1}H NMR of the (E)-2-((5-methoxy-1-methyl-1H-indol-3-yl)methylene)hydrazine-1-carbothioamide compound (**4h**).

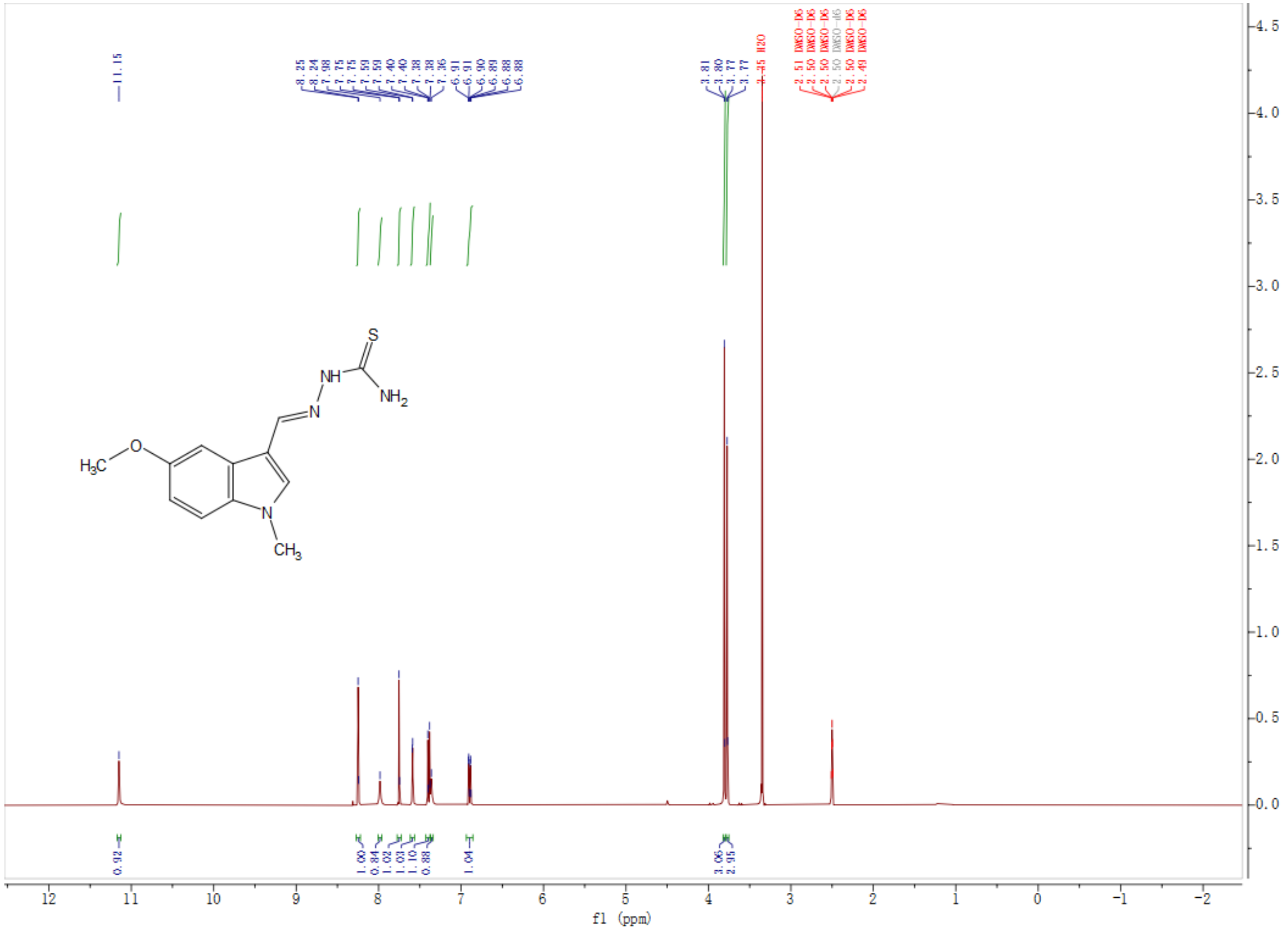

Figure S23. ^{13}C NMR of the (E)-2-((5-methoxy-1-methyl-1H-indol-3-yl)methylene)hydrazine-1-carbothioamide compound (**4h**).

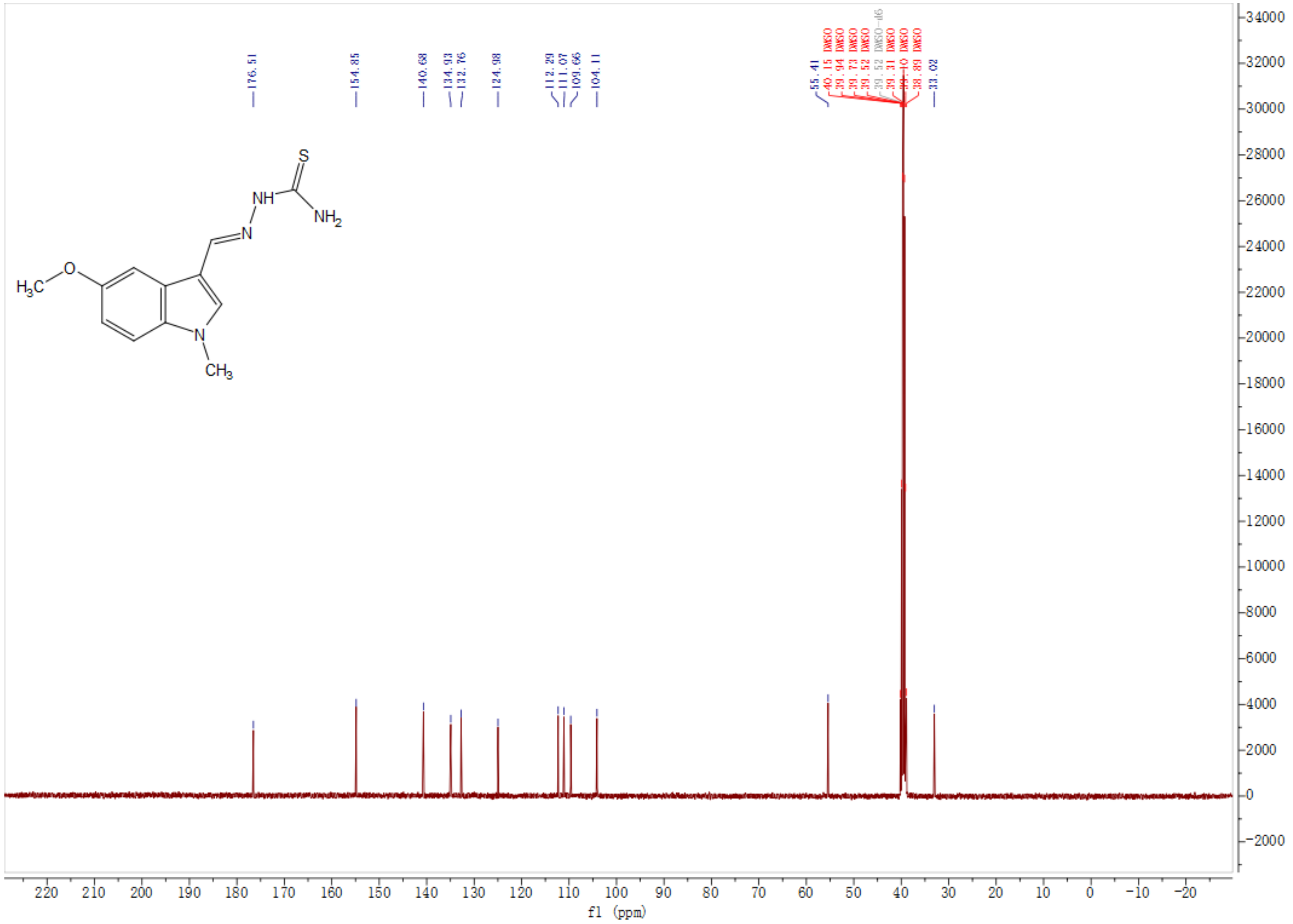

Figure S24. HRMS of the (E)-2-((5-methoxy-1-methyl-1H-indol-3-yl)methylene)hydrazine-1-carbothioamide compound (**4h**).

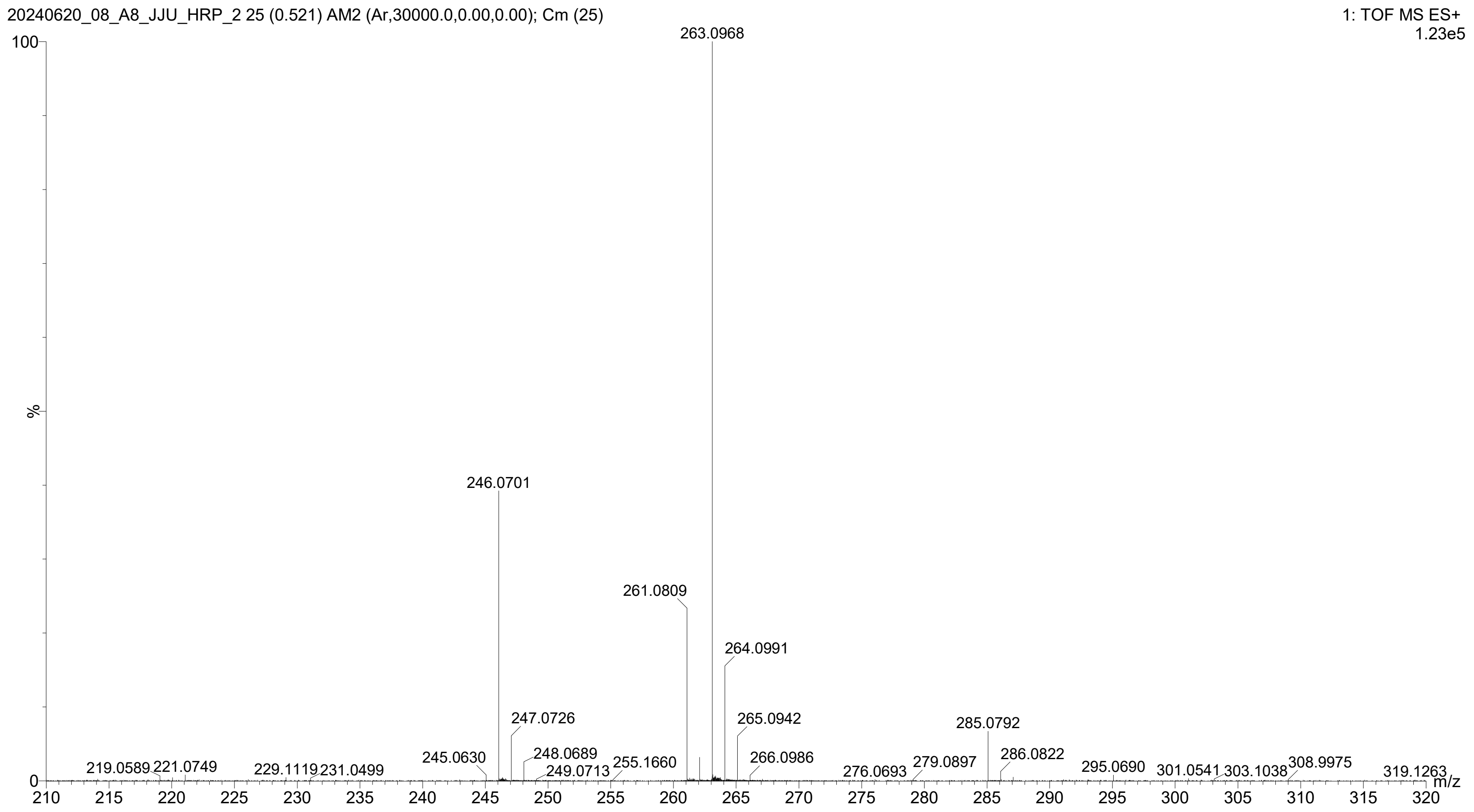

Figure S25. ^{1}H NMR of the (E)-2-((5-bromo-1-methyl-1H-indol-3-yl)methylene)hydrazine-1-carbothioamide compound (**4i**).

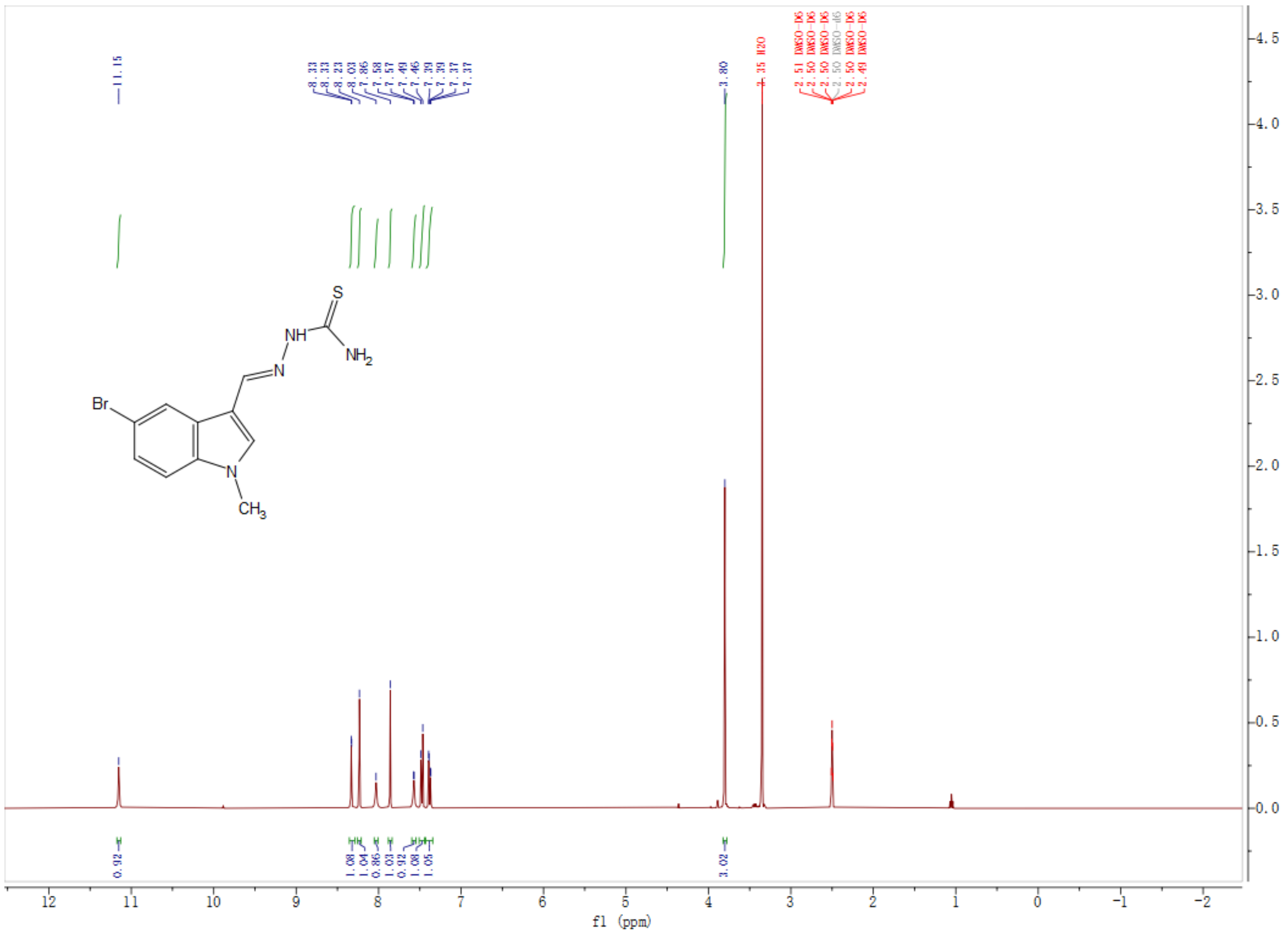

Figure S26. ^{13}C NMR of the (E)-2-((5-bromo-1-methyl-1H-indol-3-yl)methylene)hydrazine-1-carbothioamide compound (**4i**).

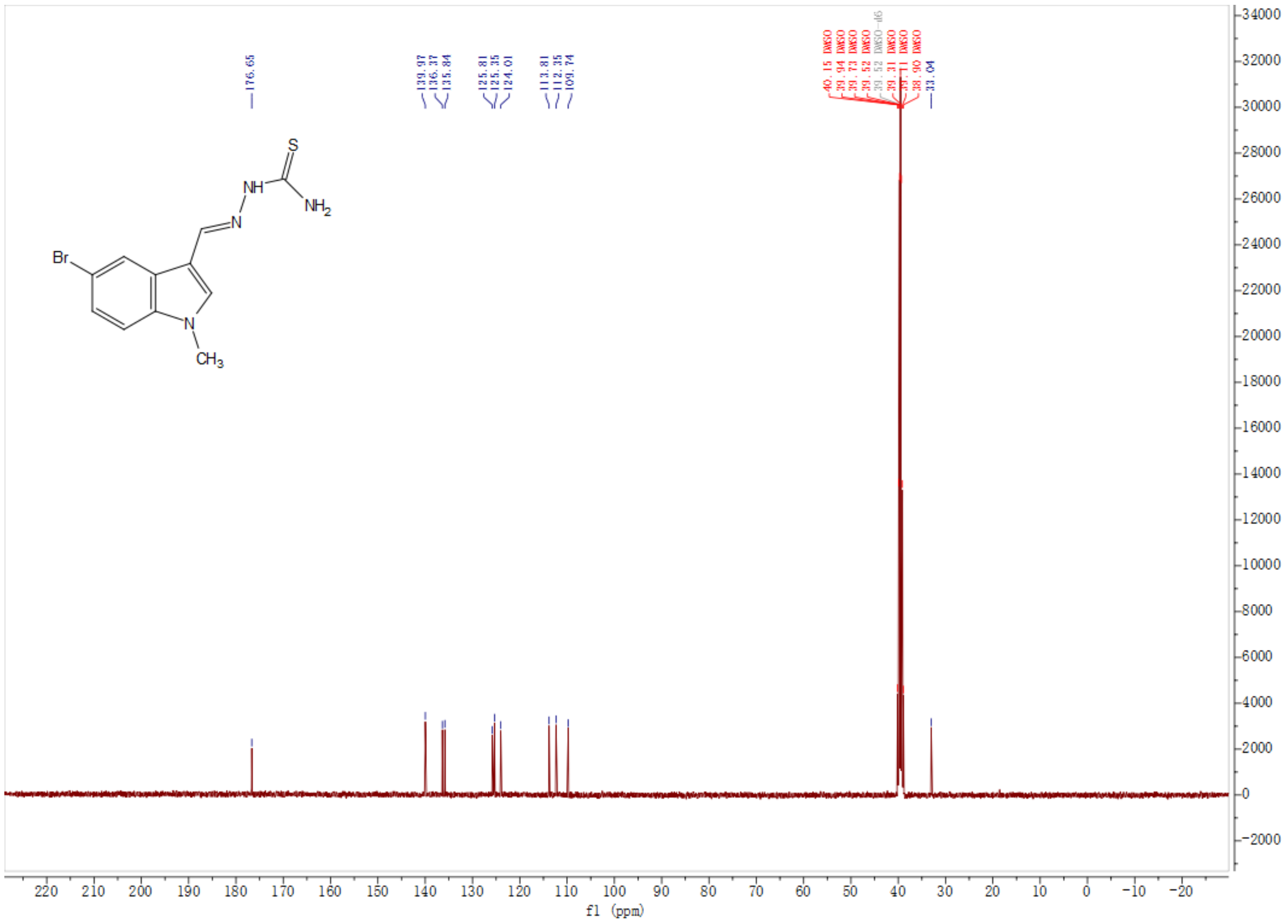

Figure S27. HRMS of the (E)-2-((5-bromo-1-methyl-1H-indol-3-yl)methylene)hydrazine-1-carbothioamide compound (**4i**).

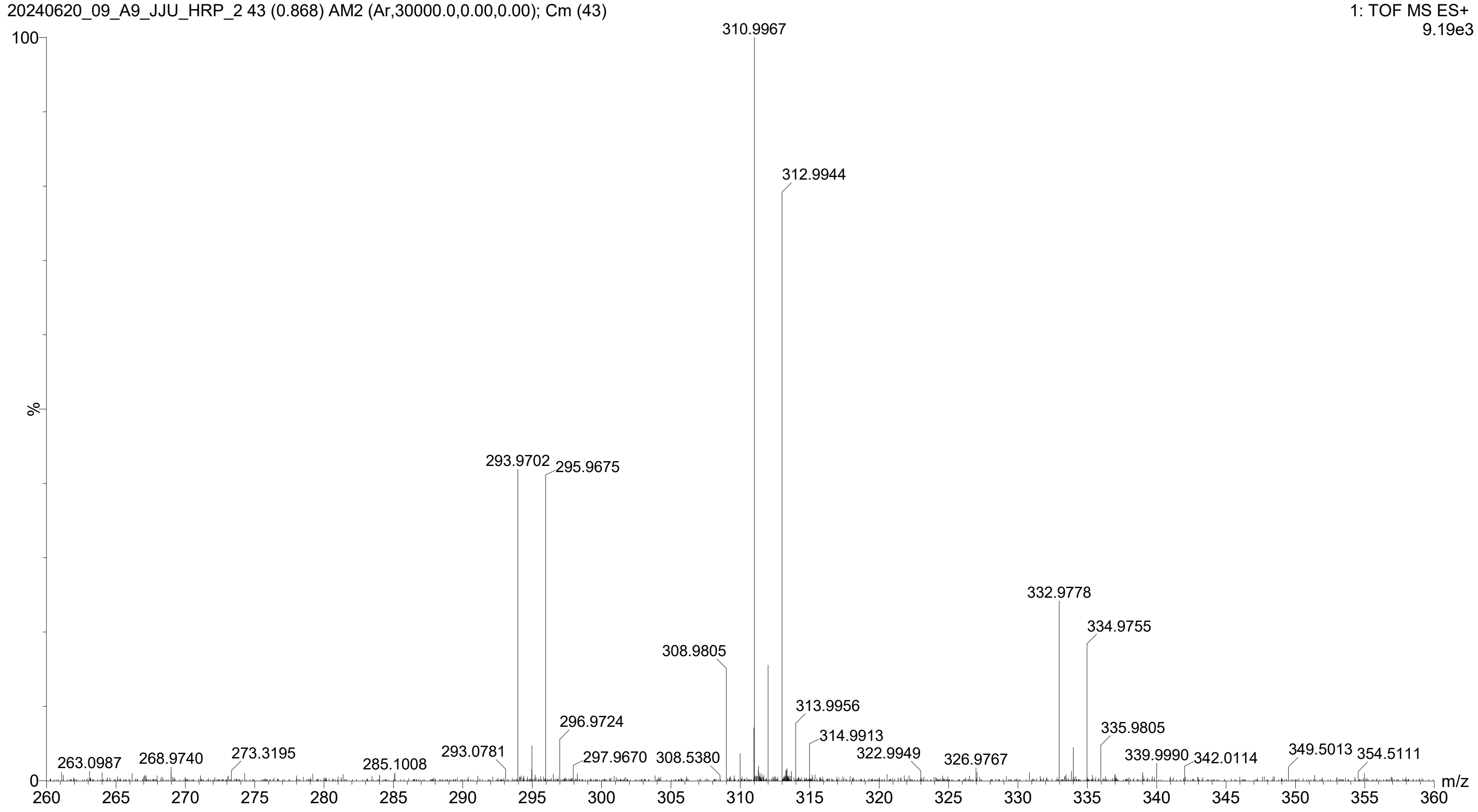

Figure S28. ^{1}H NMR of the methyl(E)-3-((2-carbamothioylhydrazineylidene)methyl)-1H-indole-5-carboxylate compound (**4j**).

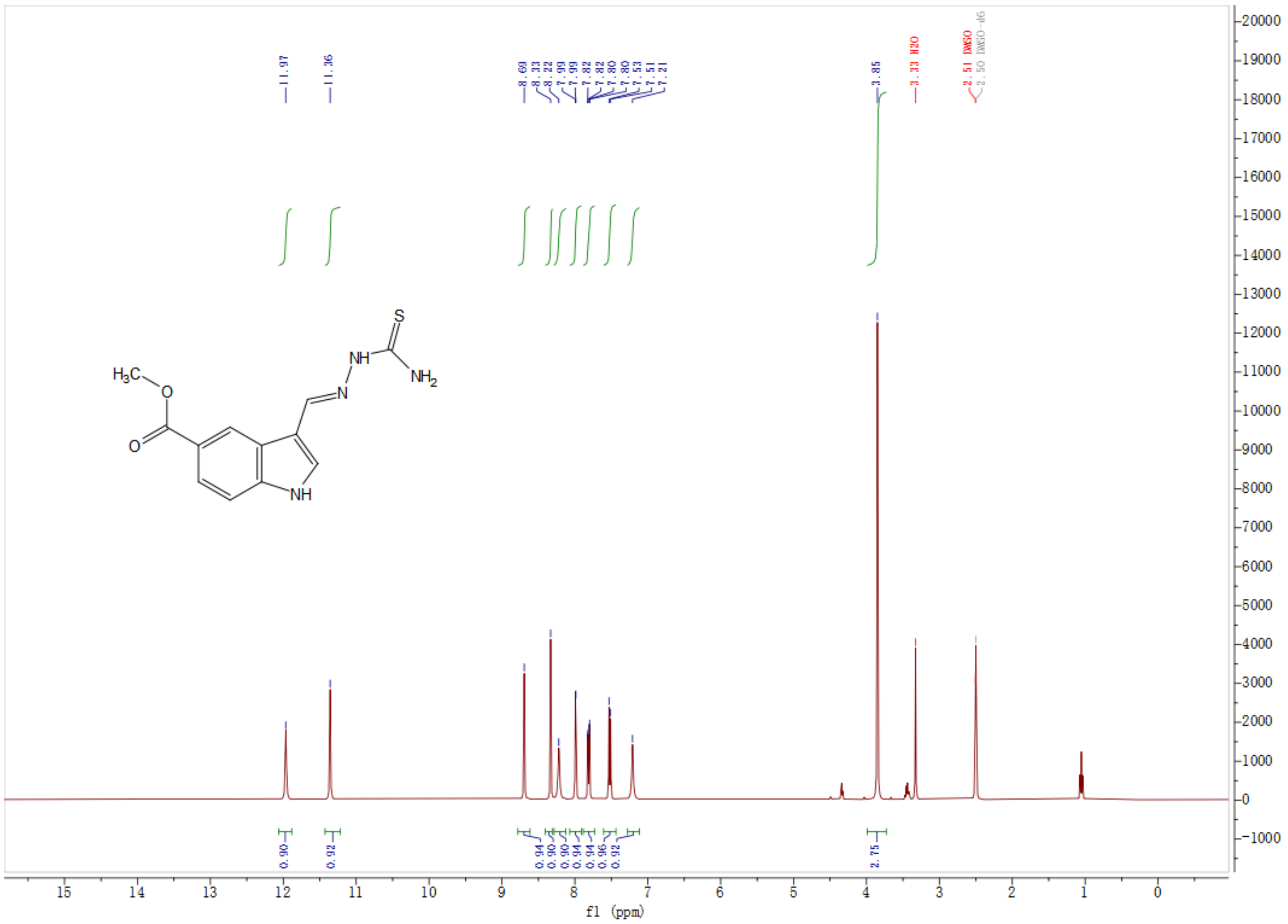

Figure S29. ^{13}C NMR of the methyl(E)-3-((2-carbamothioylhydrazineylidene)methyl)-1H-indole-5-carboxylate compound (**4j**).

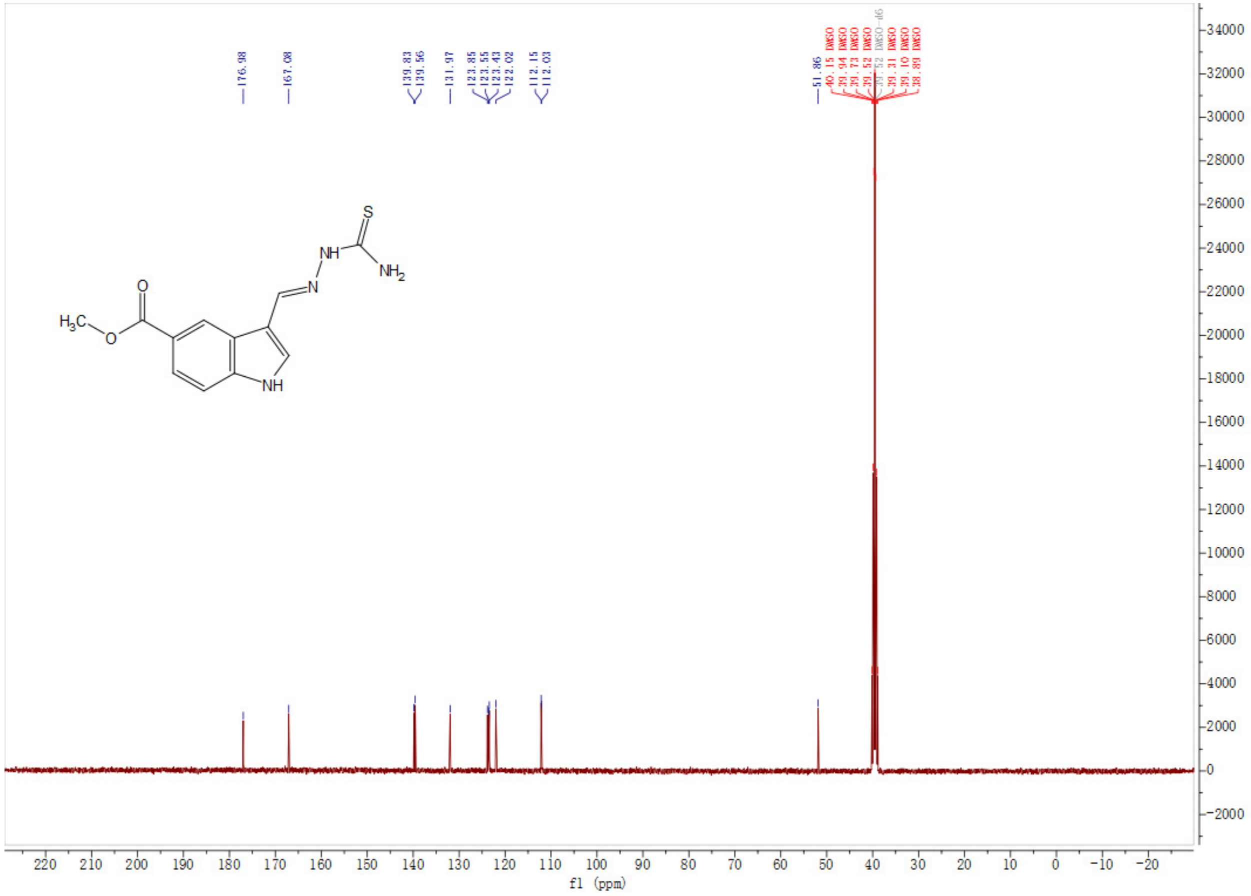

Figure S30. HRMS of the methyl(E)-3-((2-carbamothioylhydrazineylidene)methyl)-1H-indole-5-carboxylate compound (**4j**).

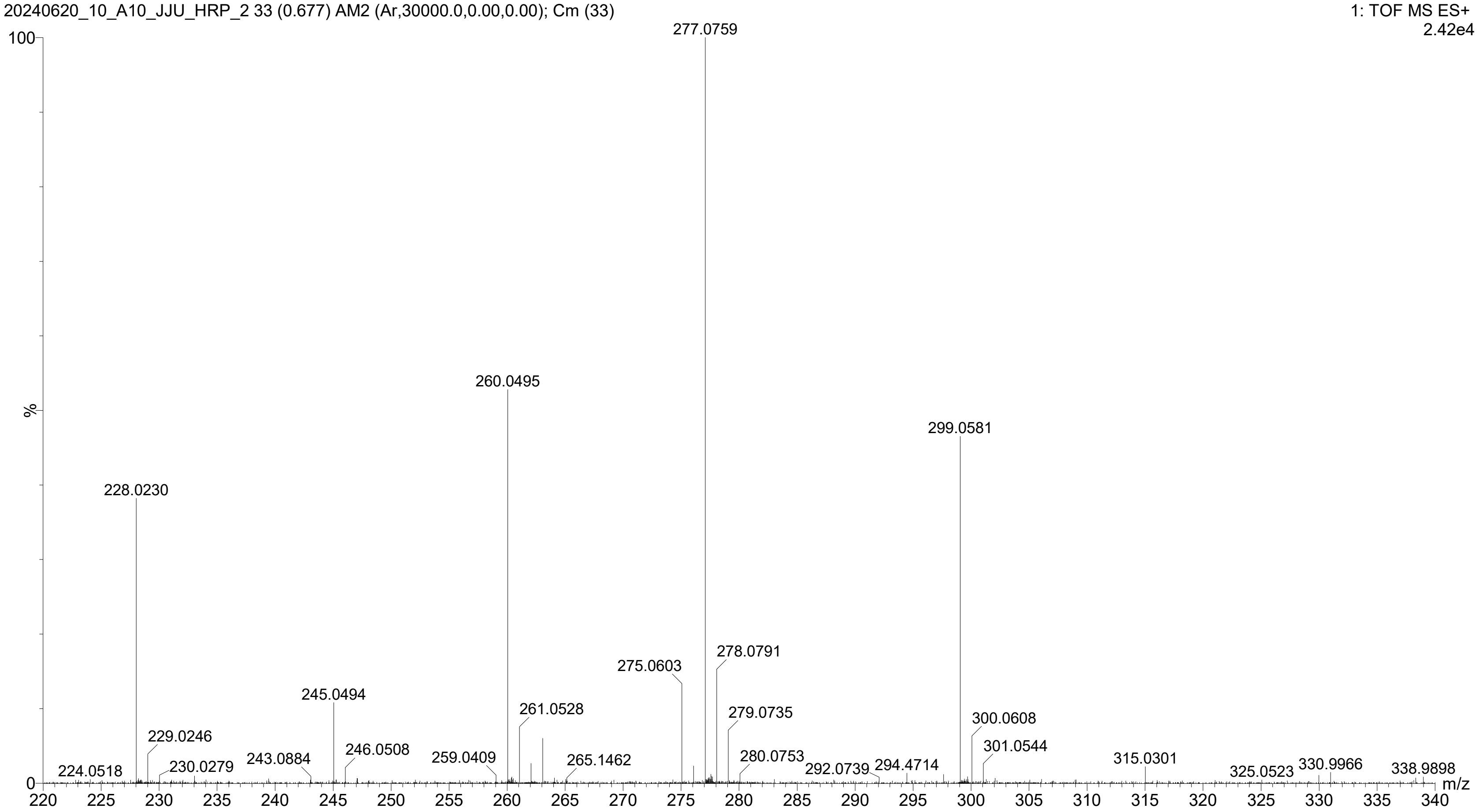

Figure S31. ^{1}H NMR of the (E)-2-((5-hydroxy-1H-indol-3-yl)methylene)hydrazine-1-carbothioamide compound (**4k**).

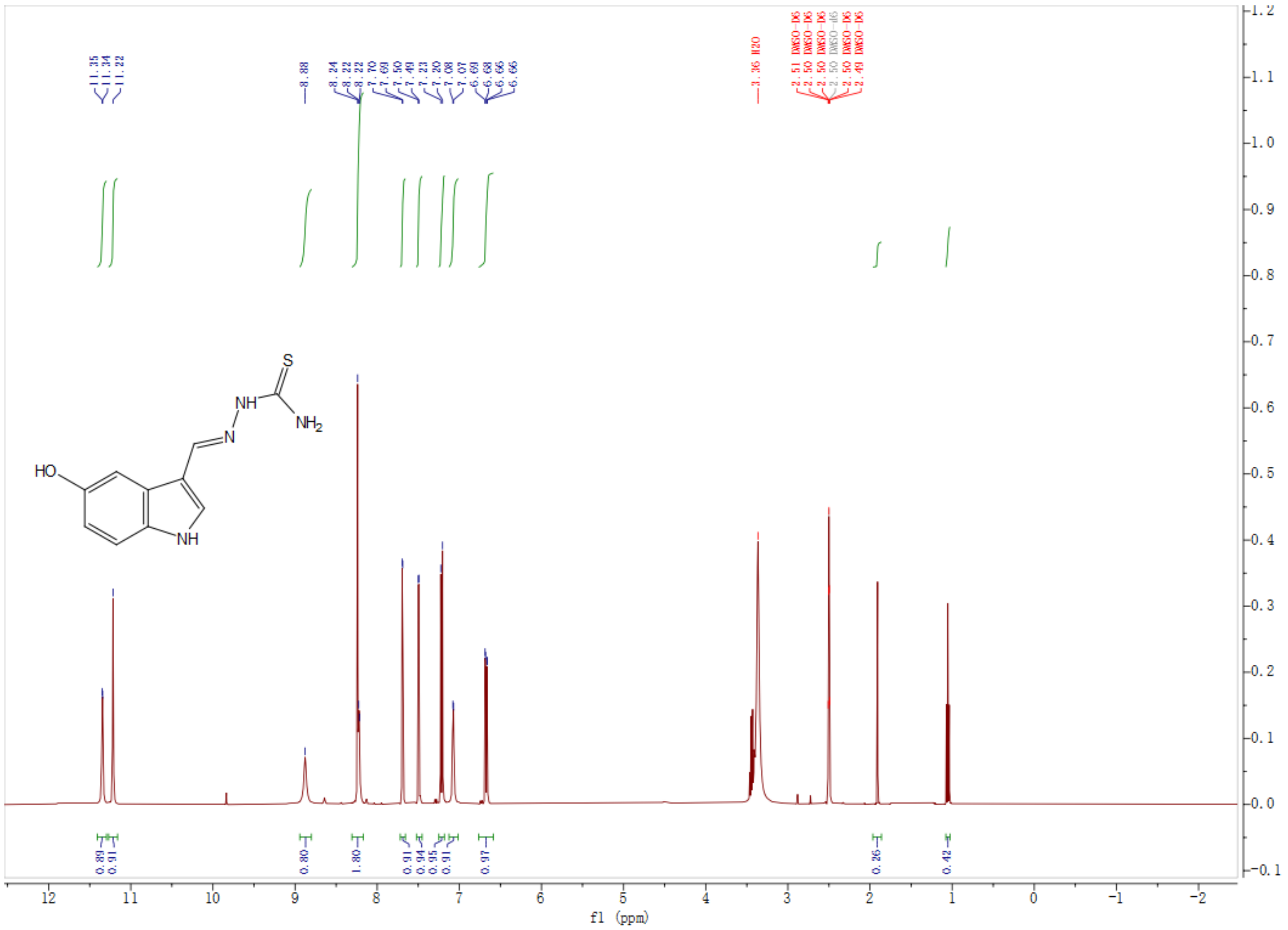

Figure S32. ^{13}C NMR of the (E)-2-((5-hydroxy-1H-indol-3-yl)methylene)hydrazine-1-carbothioamide compound (**4k**).

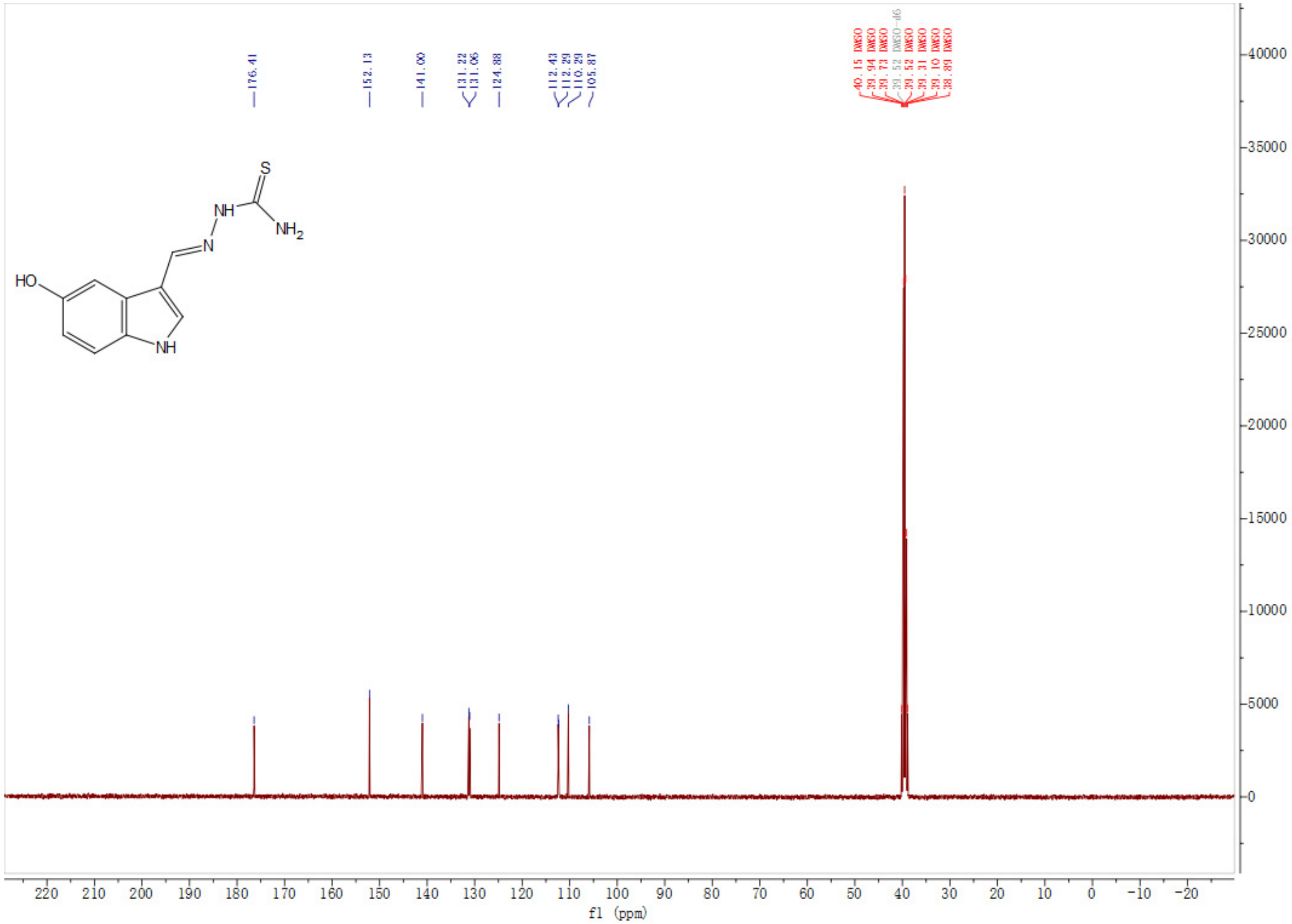

Figure S33. HRMS of the (E)-2-((5-hydroxy-1H-indol-3-yl)methylene)hydrazine-1-carbothioamide compound (**4k**).

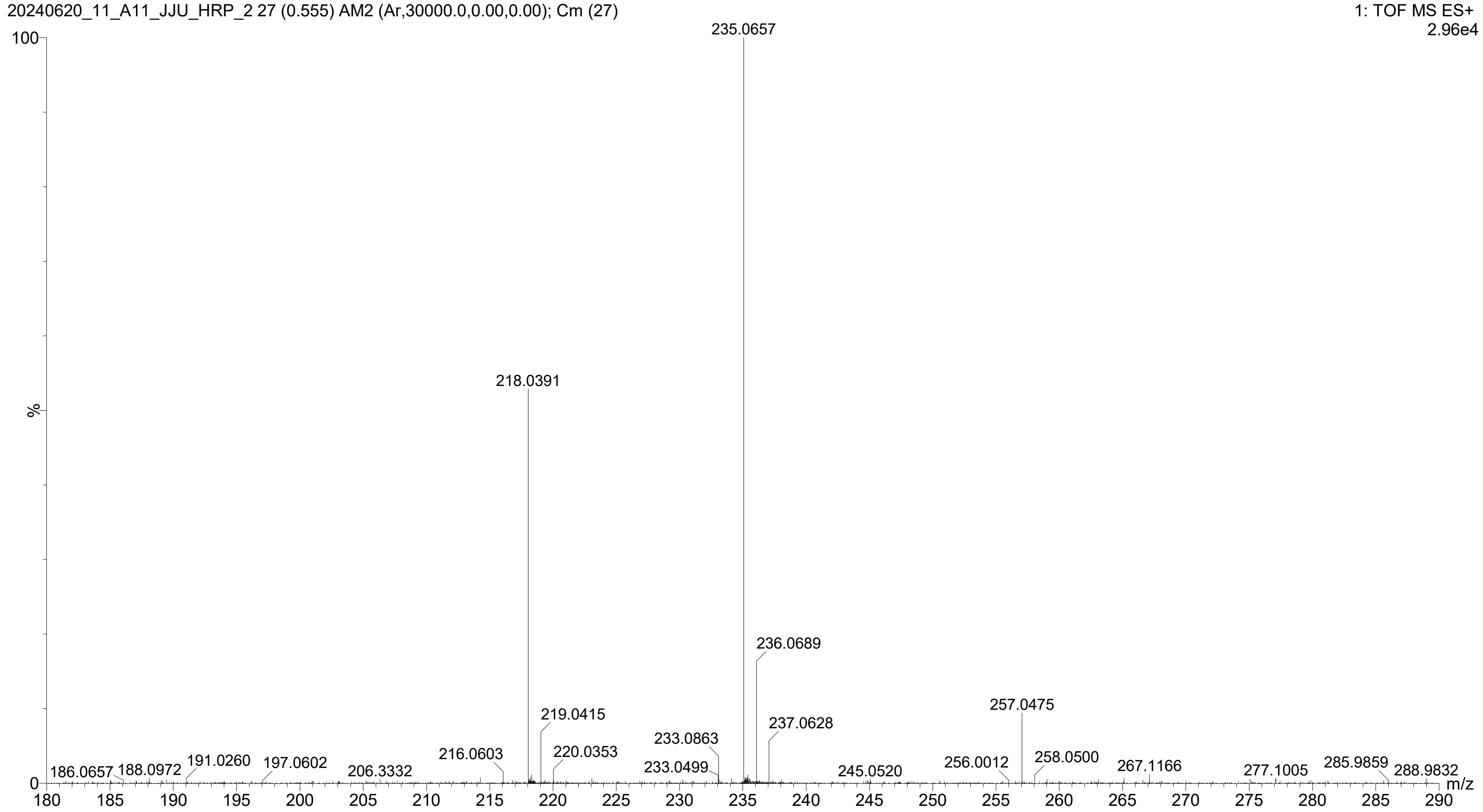

Supplement: Supplementary file 1 [file ijms-25-09636-s001.zip › ijms-3155604-supplementary.pdf]
